# Supplementary material for: Bioacoustics for in situ validation of species distribution modelling: An example with bats in Brazil
Source: PLoS One. 2021 Oct 20;16(10):e0248797. doi: 10.1371/journal.pone.0248797 (PMC8528307; doi:10.1371/journal.pone.0248797)
Supplement: S5 Table — Kruskal-Wallis test results for the comparisons between the thresholds’ performance scores is also presented. The Mann-Whitney pairwise post hoc test results are presented by letters next to average ± standard deviation (different letters indicate significant differences between groups, p < 0.05). (DOCX) [file pone.0248797.s005.docx]

**S5 Table.** Average ± standard deviation scores of accuracy, precision, sensitivity, specificity, g-mean, and f-score of the three thresholds tested for SDMs of six neotropical bat species after a field validation in northeastern Brazil using bioacoustics. Kruskal-Wallis test results for the comparisons between the thresholds' performance scores is also presented. The Mann-Whitney pairwise post hoc test results are presented by letters next to average ± standard deviation (different letters indicate significant differences between groups, p < 0.05).

| **Species** | **Validation performance metric** | **Threshold** | | | | **Kruskal-Wallis test results** | | |
| --- | --- | --- | --- | --- | --- | --- | --- | --- |
|  |  | **LPT** | **maxSSS** | **P10** | **χ^2^** | | **p** |  |
| All species | Accuracy | 0.31 ± 0.09^b^ | 0.53 ± 0.15^a^ | 0.49 ± 0.14^a^ | 62.28 | | <0.001 |  |
|  | Precision | 0.28 ± 0.11^b^ | 0.33 ± 0.12^a^ | 0.33 ± 0.11^a^ | 9.629 | | <0.05 |  |
|  | Sensitivity | 0.95 ± 0.08^a^ | 0.58 ± 0.21^c^ | 0.73 ± 0.16^b^ | 90.83 | | <0.001 |  |
|  | Specificity | 0.05 ± 0.05^c^ | 0.51 ± 0.26^a^ | 0.39 ± 0.23^b^ | 92.18 | | <0.001 |  |
|  | G-mean | 0.16 ± 0.14^b^ | 0.49 ± 0.14^a^ | 0.49 ± 0.14^a^ | 83.84 | | <0.001 |  |
|  | F-score | 0.43 ± 0.13^a,b^ | 0.40 ± 0.13^b^ | 0.44 ± 0.12^a^ | 6.244 | | <0.05 |  |
| *Noctilio leporinus* | Accuracy | 0.3 ± 0.02^c^ | 0.67 ± 0.05^a^ | 0.57 ± 0.02^b^ | 20.48 | | <0.001 |  |
|  | Precision | 0.3 ± 0^c^ | 0.47 ± 0.07^a^ | 0.38 ± 0.02^b^ | 19.86 | | <0.001 |  |
|  | Sensitivity | 1 ± 0.01^a^ | 0.59 ± 0.08^c^ | 0.74 ± 0.08^b^ | 19.42 | | <0.001 |  |
|  | Specificity | 0.01 ± 0.03^c^ | 0.71 ± 0.09^a^ | 0.5 ± 0.03^b^ | 20.48 | | <0.001 |  |
|  | G-mean | 0.05 ± 0.1^c^ | 0.64 ± 0.02^a^ | 0.61 ± 0.02^b^ | 18.36 | | <0.001 |  |
|  | F-score | 0.46 ± 0^b^ | 0.52 ± 0.02^a^ | 0.5 ± 0.03^a^ | 15.66 | | <0.001 |  |
| *Promops centralis* | Accuracy | 0.28 ± 0.01^b^ | 0.44 ± 0.14^a^ | 0.34 ± 0.02^a^ | 16.27 | | <0.001 |  |
|  | Precision | 0.19 ± 0^a^ | 0.2 ± 0.04^a^ | 0.19 ± 0.01^a^ | 0.6563 | | 0.7164 |  |
|  | Sensitivity | 0.95 ± 0.03^a^ | 0.67 ± 0.17^b^ | 0.83 ± 0.05^b^ | 16.34 | | <0.001 |  |
|  | Specificity | 0.13 ± 0.02^b^ | 0.38 ± 0.21^a^ | 0.23 ± 0.03^a^ | 16.41 | | <0.001 |  |
|  | G-mean | 0.36 ± 0.02^b^ | 0.47 ± 0.06^a^ | 0.44 ± 0.02^a^ | 15.81 | | <0.001 |  |
|  | F-score | 0.32 ± 0.01^a^ | 0.3 ± 0.02^a^ | 0.31 ± 0.01^a^ | 4.884 | | 0.0835 |  |
| *Promops nasutus* | Accuracy | 0.38 ± 0.03^b^ | 0.58 ± 0.02^a^ | 0.55 ± 0.03^a^ | 17.67 | | <0.001 |  |
|  | Precision | 0.35 ± 0.01^b^ | 0.38 ± 0.05^a^ | 0.41 ± 0.02^a^ | 9.251 | | <0.05 |  |
|  | Sensitivity | 0.97 ± 0.04^a^ | 0.42 ± 0.25^b^ | 0.74 ± 0.07^b^ | 17.78 | | <0.001 |  |
|  | Specificity | 0.08 ± 0.05^b^ | 0.67 ± 0.15^a^ | 0.45 ± 0.07^a^ | 18 | | <0.001 |  |
|  | G-mean | 0.26 ± 0.08^b^ | 0.49 ± 0.1^a^ | 0.57 ± 0.04^a^ | 15.31 | | <0.001 |  |
|  | F-score | 0.52 ± 0.01^a^ | 0.38 ± 0.14^a^ | 0.53 ± 0.02^a^ | 5.071 | | 0.0787 |  |
| *Pteronotus gymnonotus* | Accuracy | 0.42 ± 0.01^b^ | 0.48 ± 0.05^a^ | 0.44 ± 0.02^a^ | 12.31 | | <0.05 |  |
|  | Precision | 0.41 ± 0^a^ | 0.37 ± 0.07^a^ | 0.42 ± 0.01^a^ | 1.046 | | 0.5895 |  |
|  | Sensitivity | 1 ± 0^a^ | 0.47 ± 0.34^c^ | 0.91 ± 0.03^b^ | 19.42 | | <0.001 |  |
|  | Specificity | 0.01 ± 0.02^c^ | 0.49 ± 0.28^a^ | 0.11 ± 0.02^b^ | 19.57 | | <0.001 |  |
|  | G-mean | 0.06 ± 0.09^c^ | 0.39 ± 0.06^a^ | 0.31 ± 0.03^b^ | 18.87 | | <0.001 |  |
|  | F-score | 0.58 ± 0^a^ | 0.38 ± 0.16^b^ | 0.57 ± 0.01^b^ | 10.28 | | <0.05 |  |
| *Pteronotus personatus* | Accuracy | 0.19 ± 0.02^c^ | 0.42 ± 0.11^a^ | 0.32 ± 0.04^b^ | 18.12 | | <0.001 |  |
|  | Precision | 0.15 ± 0.01^a^ | 0.15 ± 0.03^a^ | 0.15 ± 0.01^a^ | 2.205 | | 0.3301 |  |
|  | Sensitivity | 0.82 ± 0.11^a^ | 0.55 ± 0.2^b^ | 0.7 ± 0.06^b^ | 12.88 | | <0.001 |  |
|  | Specificity | 0.07 ± 0.04^a^ | 0.4 ± 0.17^b^ | 0.25 ± 0.06^b^ | 17.78 | | <0.001 |  |
|  | G-mean | 0.22 ± 0.08^b^ | 0.43 ± 0.07^a^ | 0.41 ± 0.04^a^ | 16.64 | | <0.001 |  |
|  | F-score | 0.25 ± 0.02^a^ | 0.23 ± 0.05^a^ | 0.25 ± 0.01^a^ | 0.9237 | | 0.6293 |  |
| *Saccopteryx leptura* | Accuracy | 0.2 ± 0.02^b^ | 0.69 ± 0.17^a^ | 0.74 ± 0.01^a^ | 15.38 | | <0.001 |  |
|  | Precision | 0.17 ± 0.01^b^ | 0.38 ± 0.1^a^ | 0.4 ± 0.01^a^ | 14.03 | | <0.001 |  |
|  | Sensitivity | 0.89 ± 0.03^a^ | 0.68 ± 0.07^b^ | 0.73 ± 0.02^b^ | 16.34 | | <0.001 |  |
|  | Specificity | 0.04 ± 0.03^b^ | 0.69 ± 0.22^a^ | 0.75 ± 0.01^a^ | 15.36 | | <0.001 |  |
|  | G-mean | 0.16 ± 0.09^c^ | 0.67 ± 0.12^b^ | 0.74 ± 0.01^a^ | 18.36 | | <0.001 |  |
|  | F-score | 0.29 ± 0.01^b^ | 0.47 ± 0.08^a^ | 0.51 ± 0.02^a^ | 13.03 | | <0.05 |  |
