# Supplementary material for: Bioacoustics for in situ validation of species distribution modelling: An example with bats in Brazil
Source: PLoS One. 2021 Oct 20;16(10):e0248797. doi: 10.1371/journal.pone.0248797 (PMC8528307; doi:10.1371/journal.pone.0248797)
Supplement: S1 File — Maps made with Natural Earth. Free vector and raster map data @ naturalearthdata.com. (PDF) [file pone.0248797.s006.pdf]

Noctilio leporinus

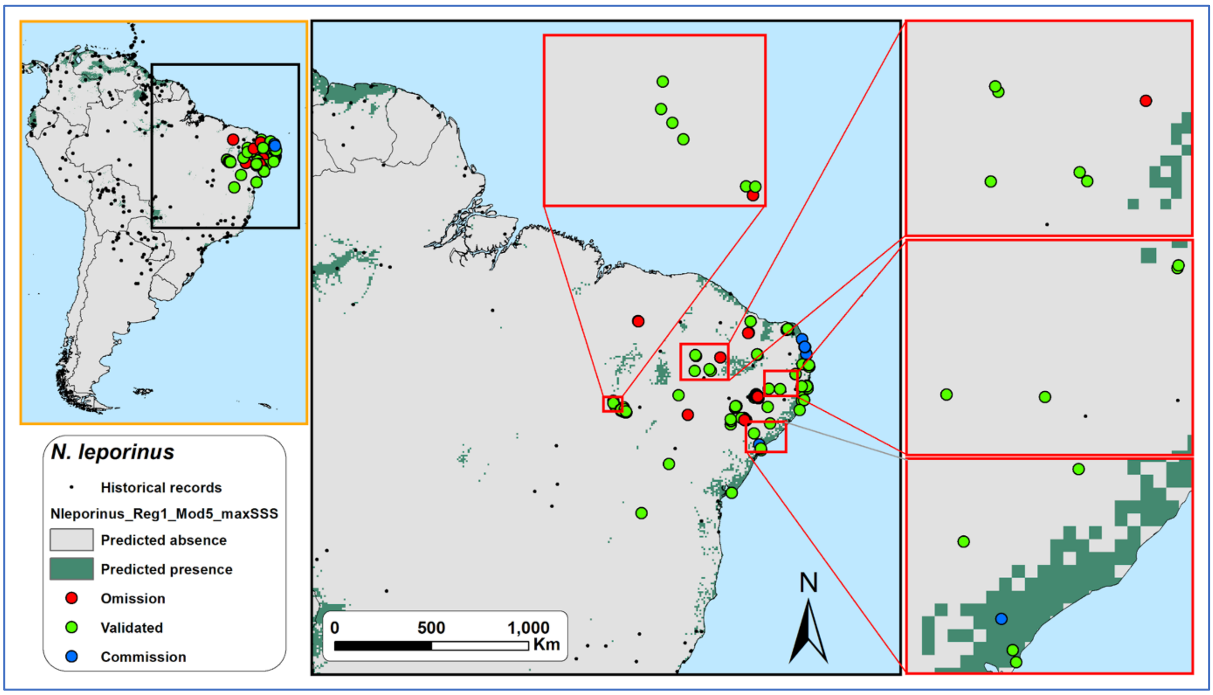

Figure A - Field validation results for the binary maps with the highest accuracy, precision, and specificity scores for *Noctilio leporinus* in northeastern Brazil. 'Omission' points represent locations where the model did not predict the species occurrence, but the species was detected during the acoustics monitoring; 'Validated' points represent locations where the model predict the species occurrence and the species was detected during the acoustics monitoring or locations where the model did not predict the species occurrence, and the species was detected during the acoustics monitoring; 'commission' points represent locations where the model predict the species occurrence but the species was not detected during the acoustics monitoring. Made with Natural Earth. Free vector and raster map data @ naturalearthdata.com.

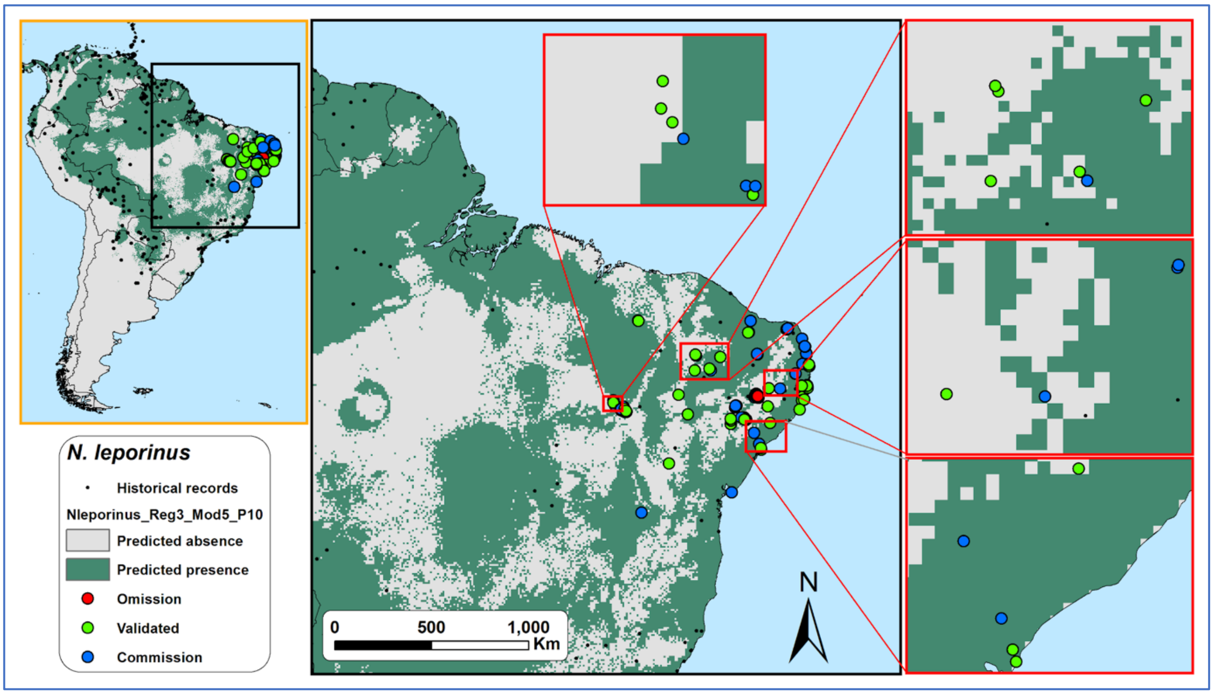

Figure B - Field validation results for the binary maps with the highest f-score score for *Noctilio leporinus* in northeastern Brazil. See Fig. A caption for the omission, validation and commission points explanation.

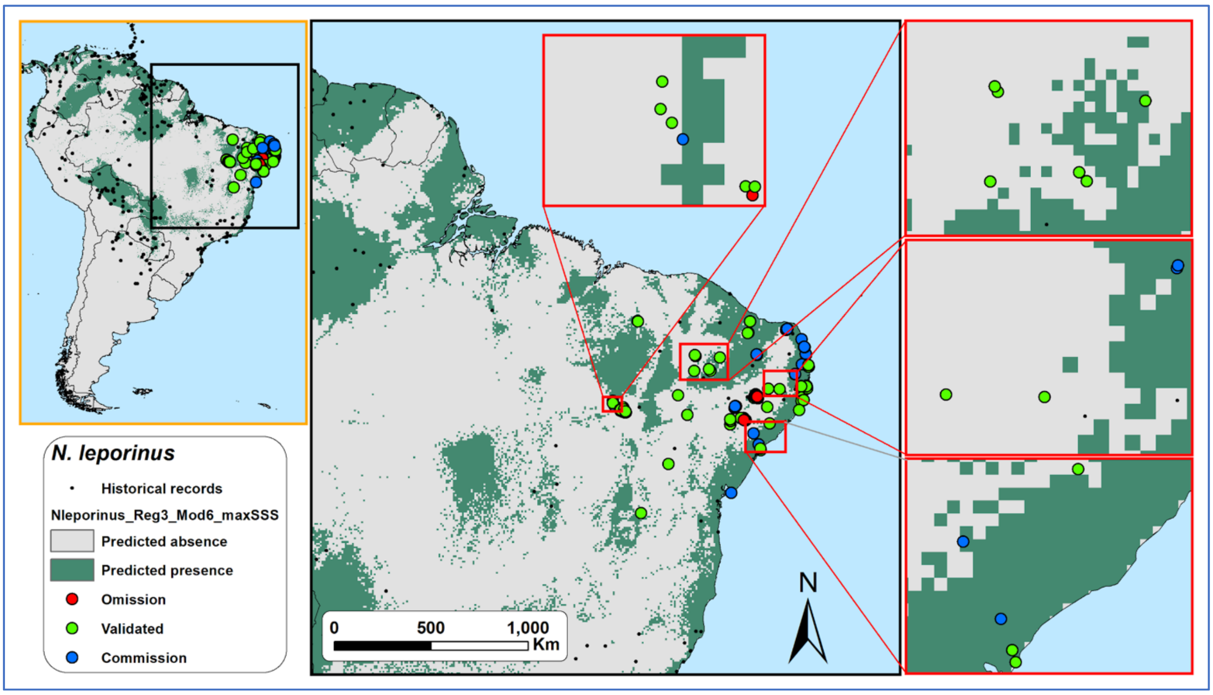

Figure C - Field validation results for the binary maps with the highest g-mean score for *Noctilio leporinus* in northeastern Brazil. See Fig. A caption for the omission, validation and commission points explanation.

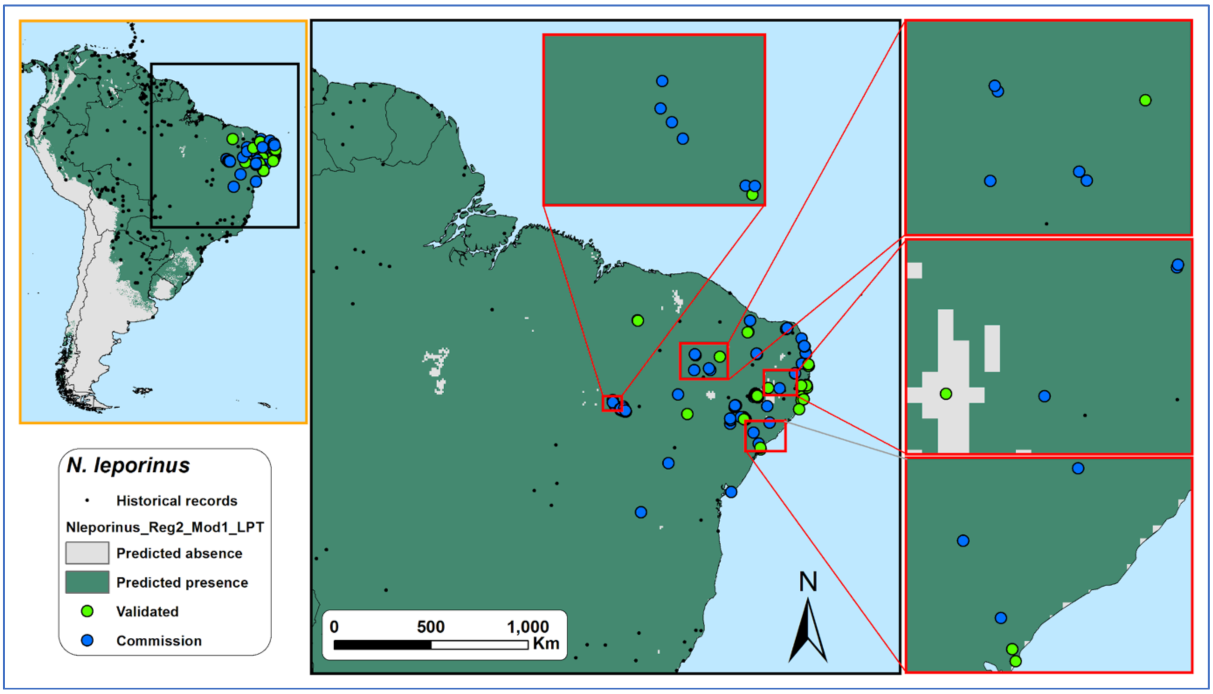

Figure D - Field validation results for the binary maps with the highest sensitivity score for *Noctilio leporinus* in northeastern Brazil. See Fig. A caption for the omission, validation and commission points explanation.

Promops centralis

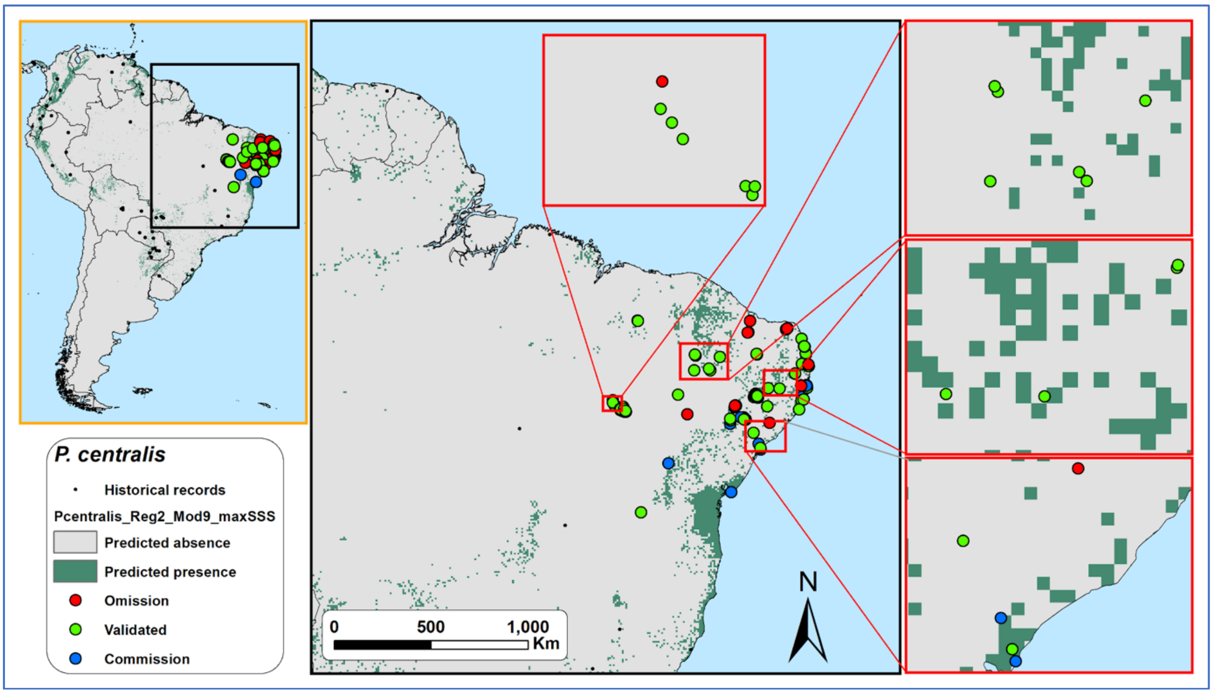

Figure E - Field validation results for the binary maps with the highest accuracy, precision, and specificity scores for *Promops centralis* in northeastern Brazil. See Fig. A caption for the omission, validation and commission points explanation.

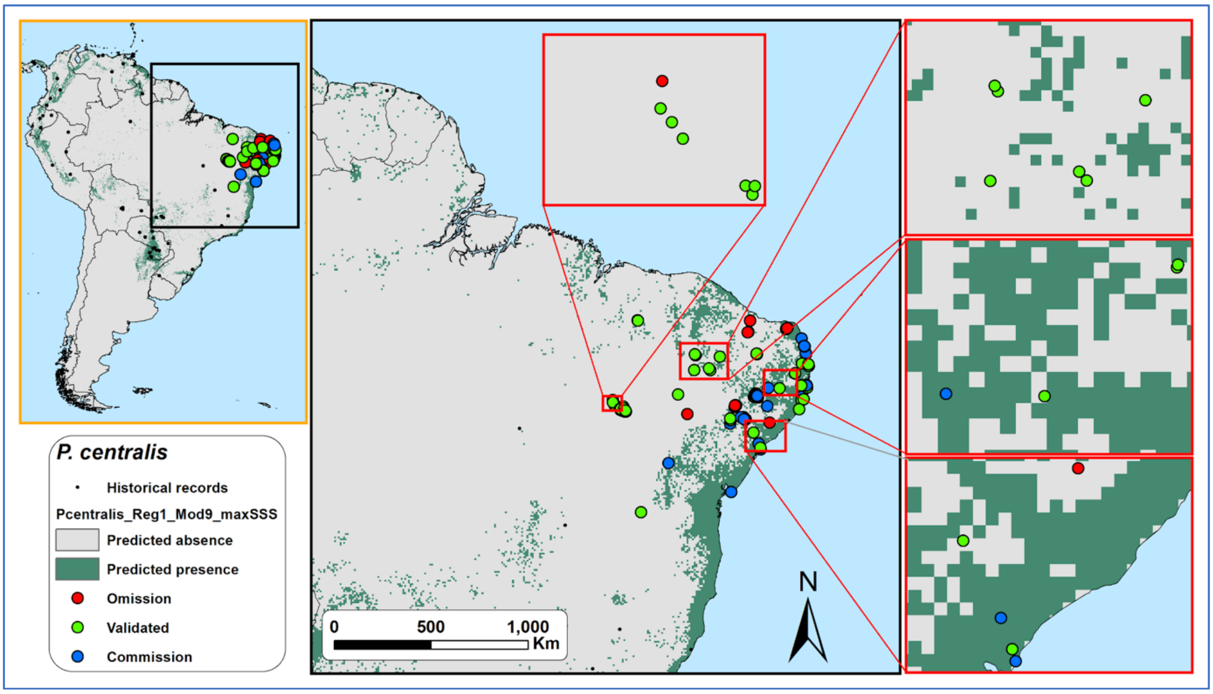

Figure F - Field validation results for the binary maps with the highest g-mean score for *Promops centralis* in northeastern Brazil. See Fig. A caption for the omission, validation and commission points explanation.

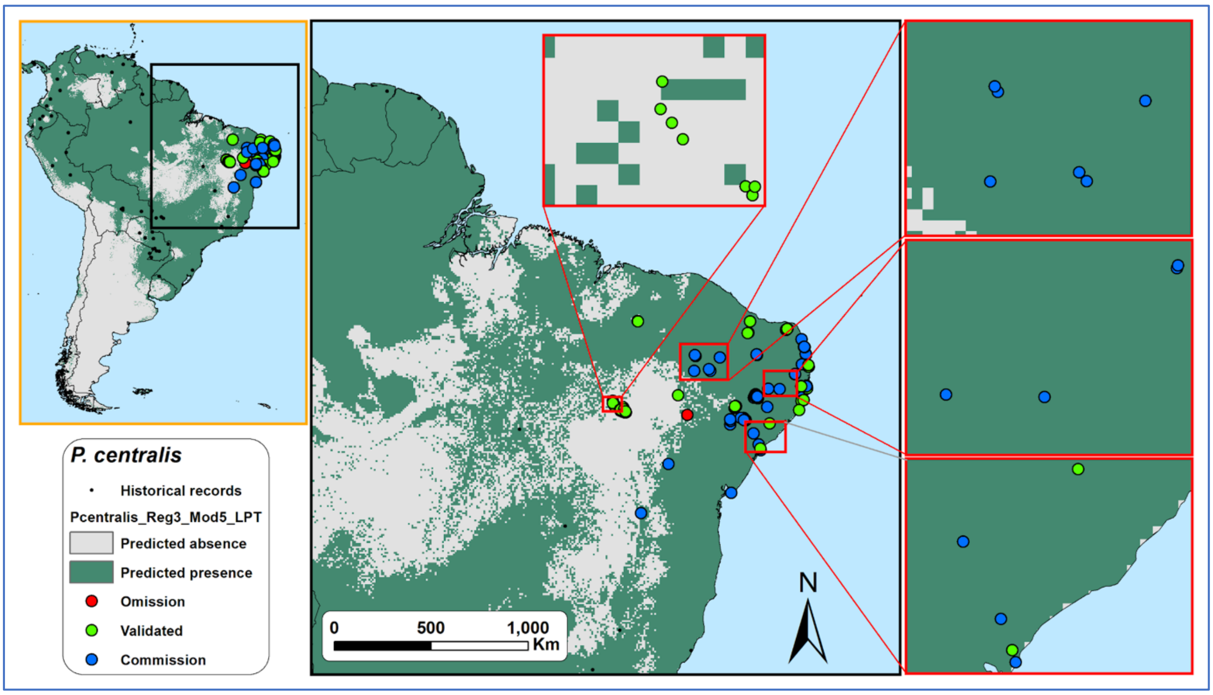

Figure G - Field validation results for the binary maps with the highest sensitivity and f-score scores for *Promops centralis* in northeastern Brazil. See Fig. A caption for the omission, validation and commission points explanation.

Pteronotus gymnonotus

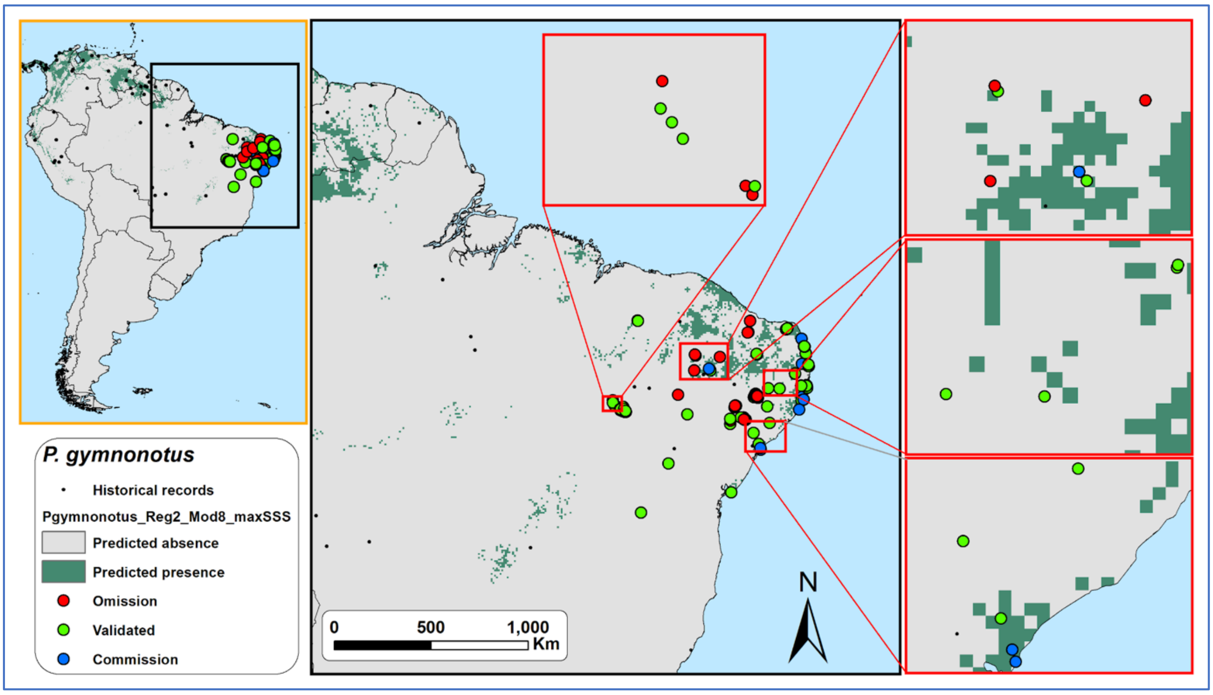

Figure H - Field validation results for the binary maps with the highest accuracy, precision, and specificity scores for *Pteronotus gymnonotus* in northeastern Brazil. See Fig. A caption for the omission, validation and commission points explanation.

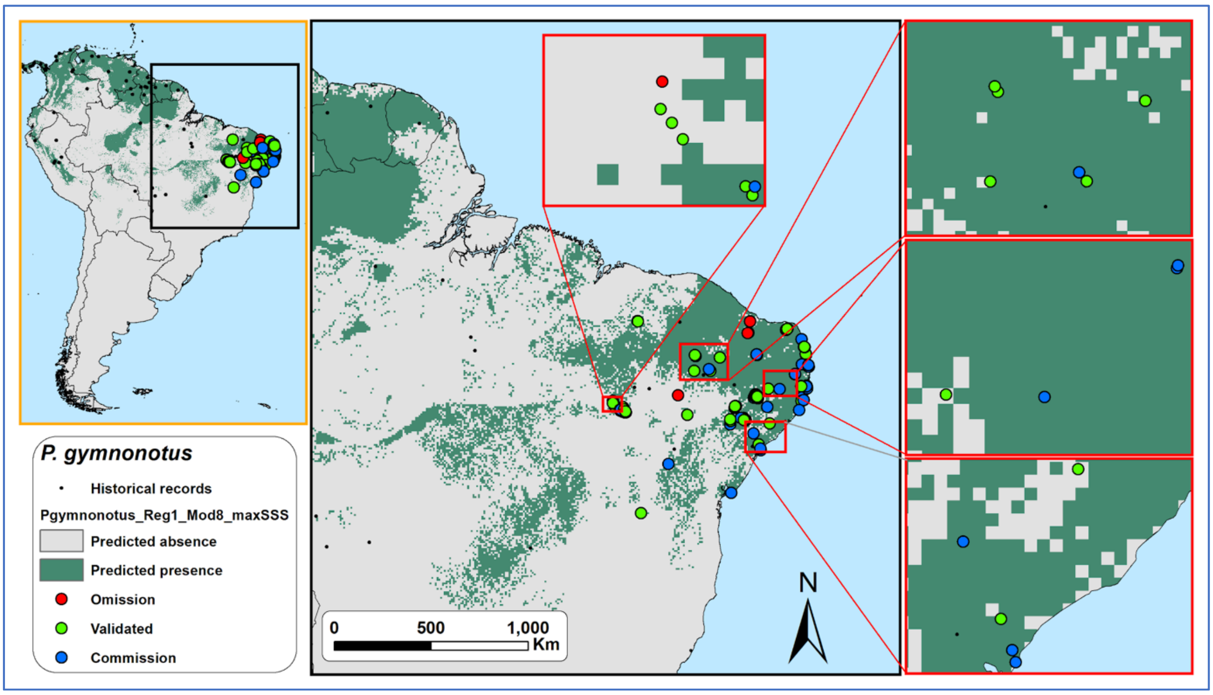

Figure I - Field validation results for the binary maps with the highest g-mean and f-score scores for *Pteronotus gymnonotus* in northeastern Brazil. See Fig. A caption for the omission, validation and commission points explanation.

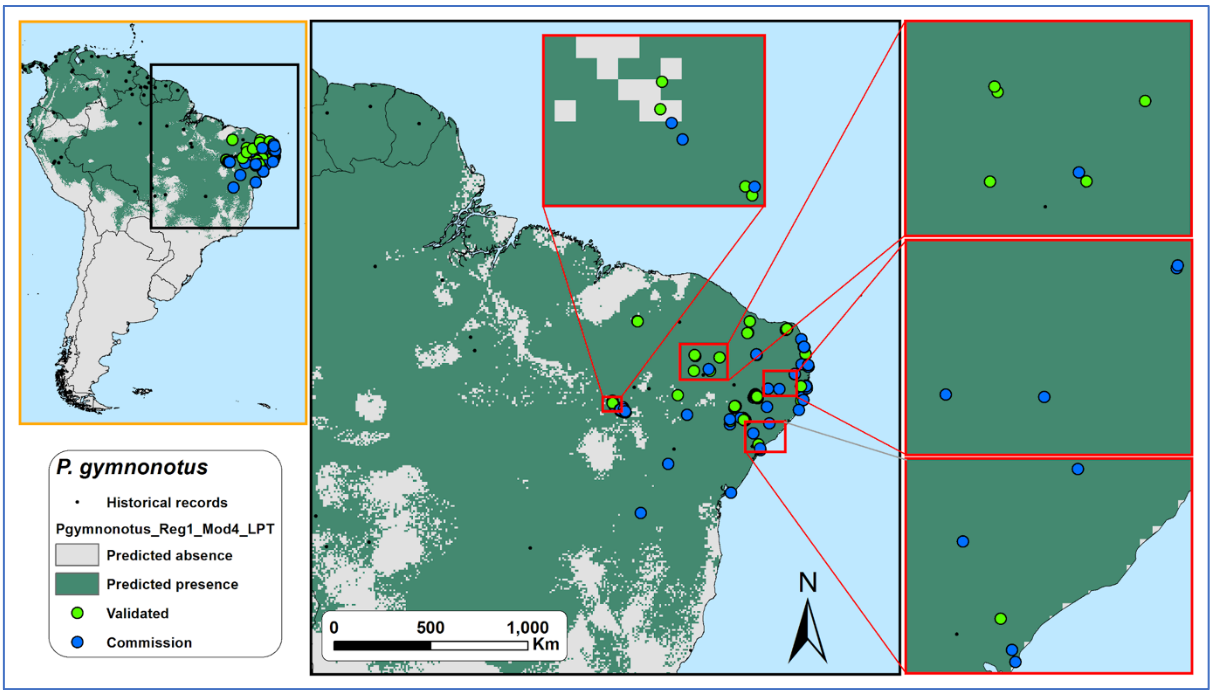

Figure J - Field validation results for the binary maps with the highest sensitivity score for *Pteronotus gymnonotus* in northeastern Brazil. See Fig. A caption for the omission, validation and commission points explanation.

Promops nasutus

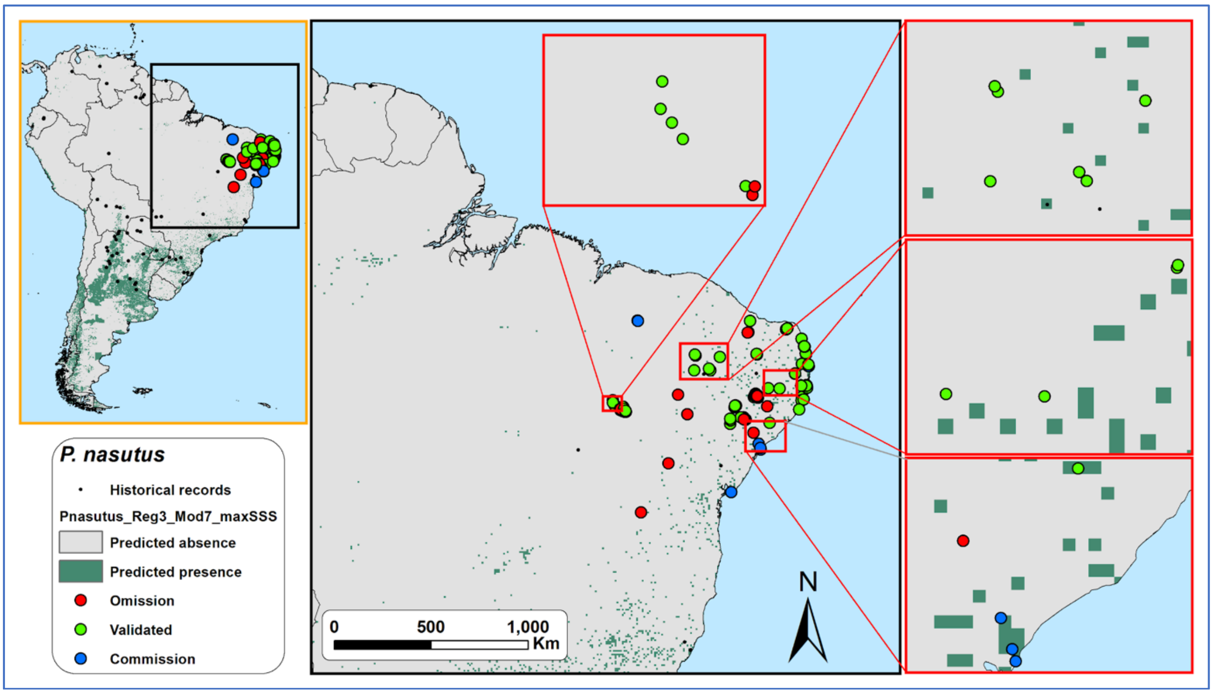

Figure K - Field validation results for the binary maps with the highest accuracy and specificity scores for *Promops nasutus* in northeastern Brazil. See Fig. A caption for the omission, validation and commission points explanation.

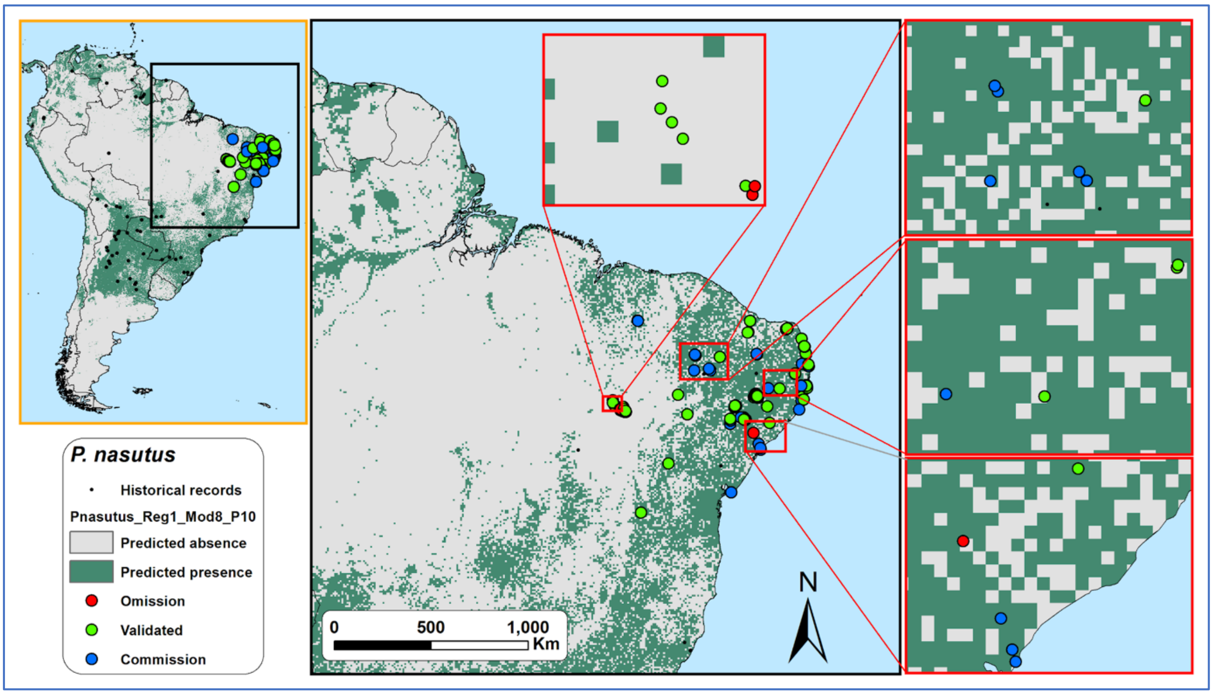

Figure L - Field validation results for the binary maps with the highest f-score score for *Promops nasutus* in northeastern Brazil. See Fig. A caption for the omission, validation and commission points explanation.

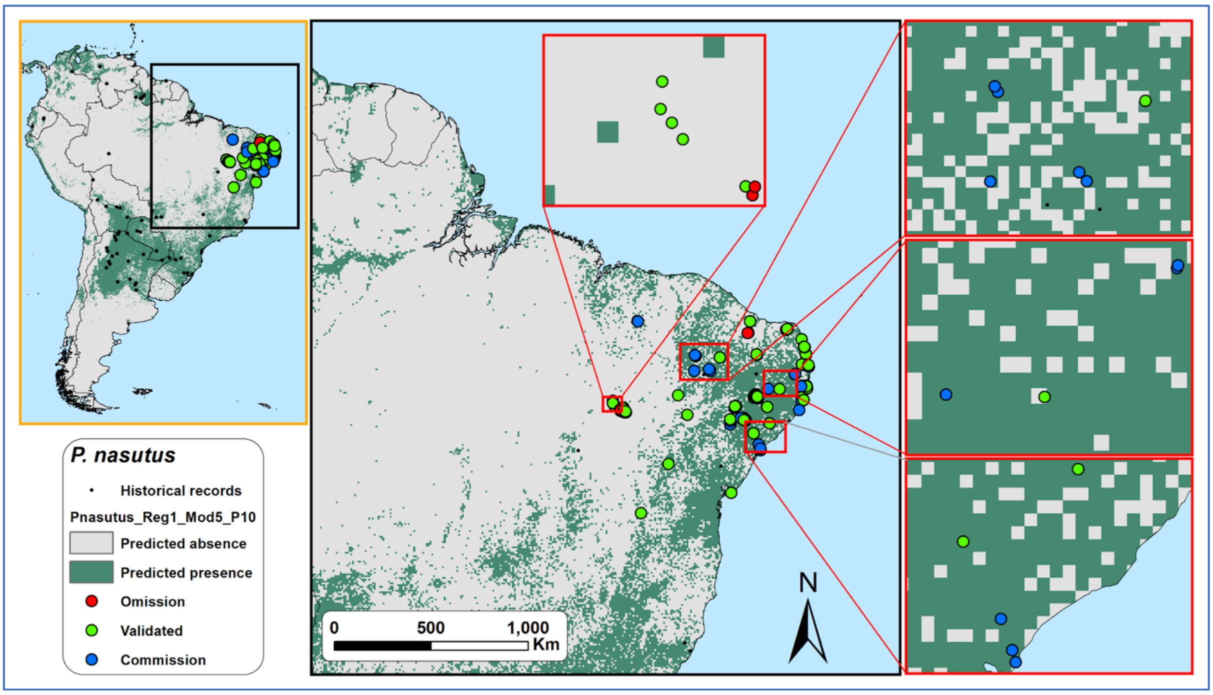

Figure M - Field validation results for the binary maps with the highest g-mean score for *Promops nasutus* in northeastern Brazil. See Fig. A caption for the omission, validation and commission points explanation.

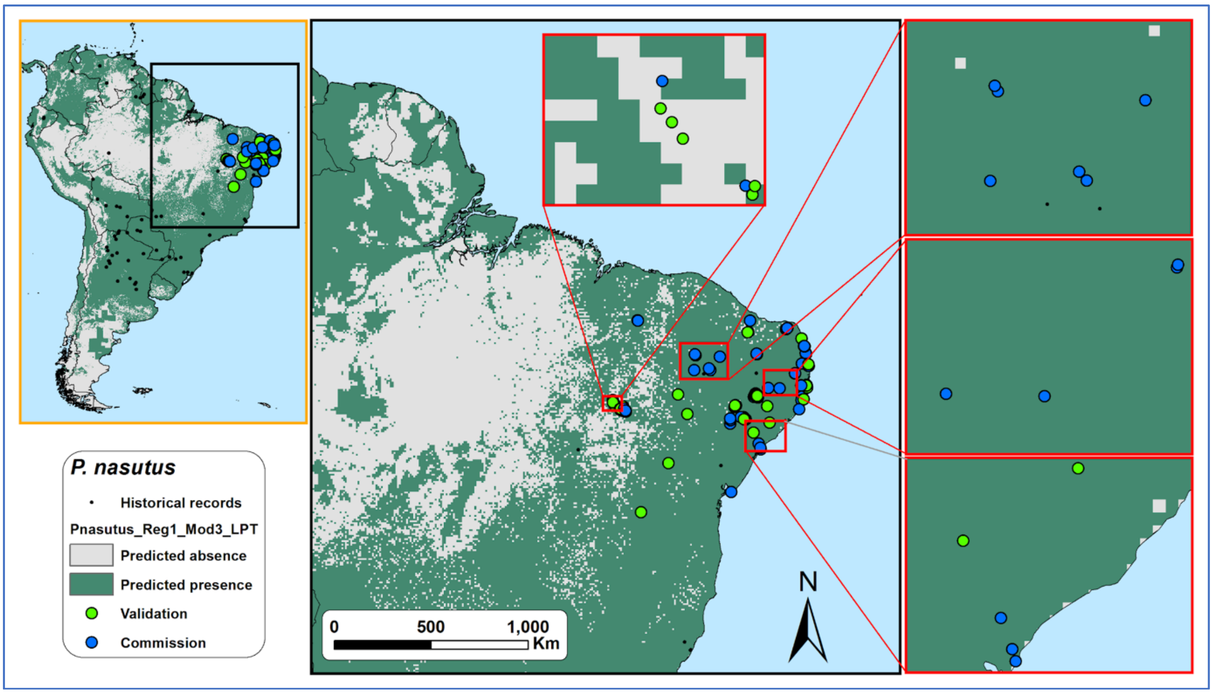

Figure N - Field validation results for the binary maps with the highest sensitivity score for *Promops nasutus* in northeastern Brazil. See Fig. A caption for the omission, validation and commission points explanation.

Pteronotus personatus

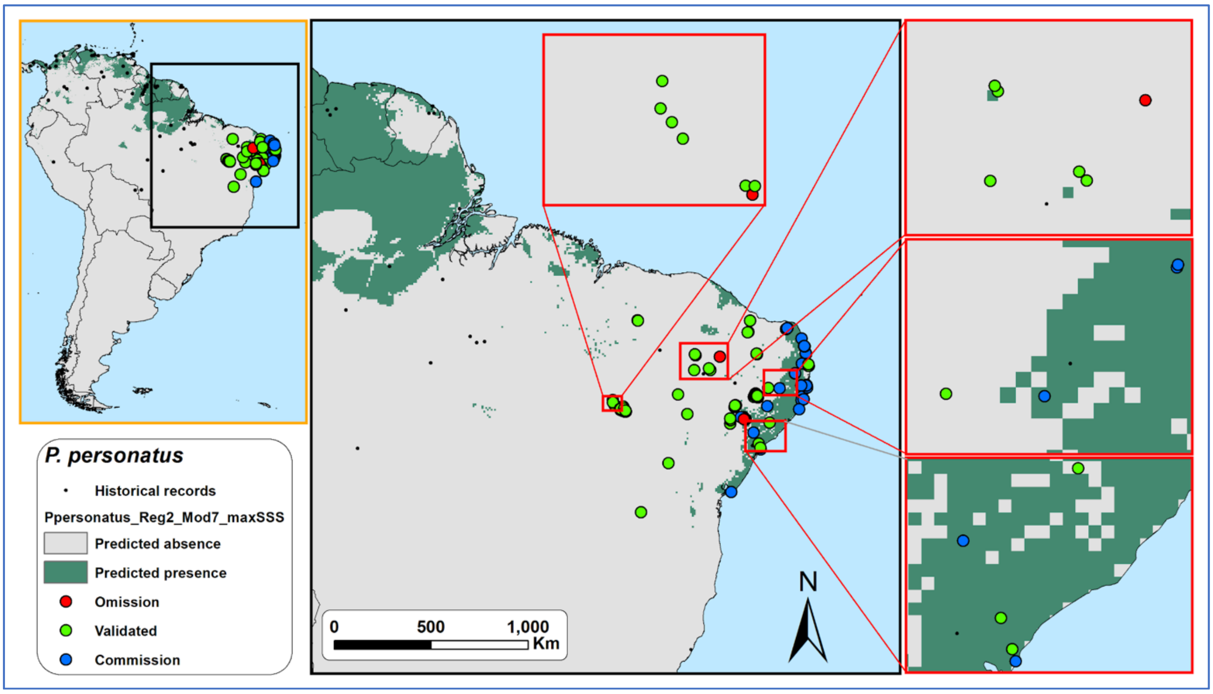

Figure O - Field validation results for the binary maps with the highest accuracy and specificity scores for *Pteronotus personatus* in northeastern Brazil. See Fig. A caption for the omission, validation and commission points explanation.

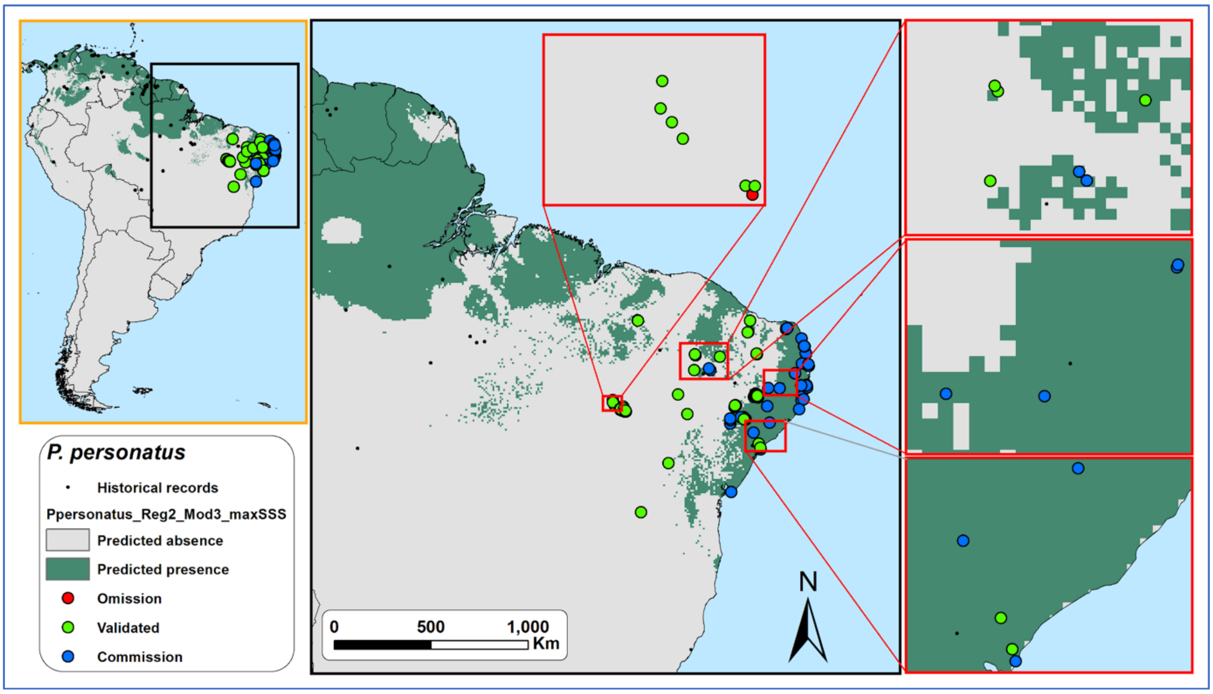

Figure P - Field validation results for the binary maps with the highest precision and g-mean scores for *Pteronotus personatus* in northeastern Brazil. See Fig. A caption for the omission, validation and commission points explanation.

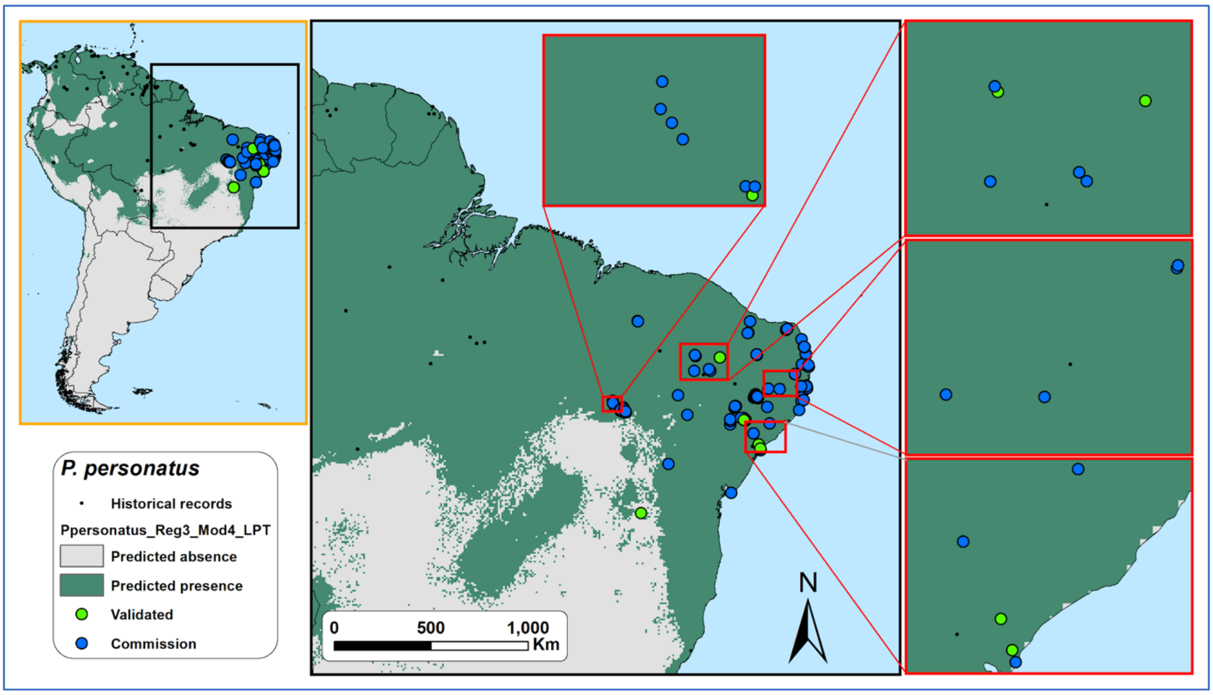

Figure Q - Field validation results for the binary maps with the highest sensitivity and f-score scores for *Pteronotus personatus* in northeastern Brazil. See Fig. A caption for the omission, validation and commission points explanation.

Saccopteryx leptura

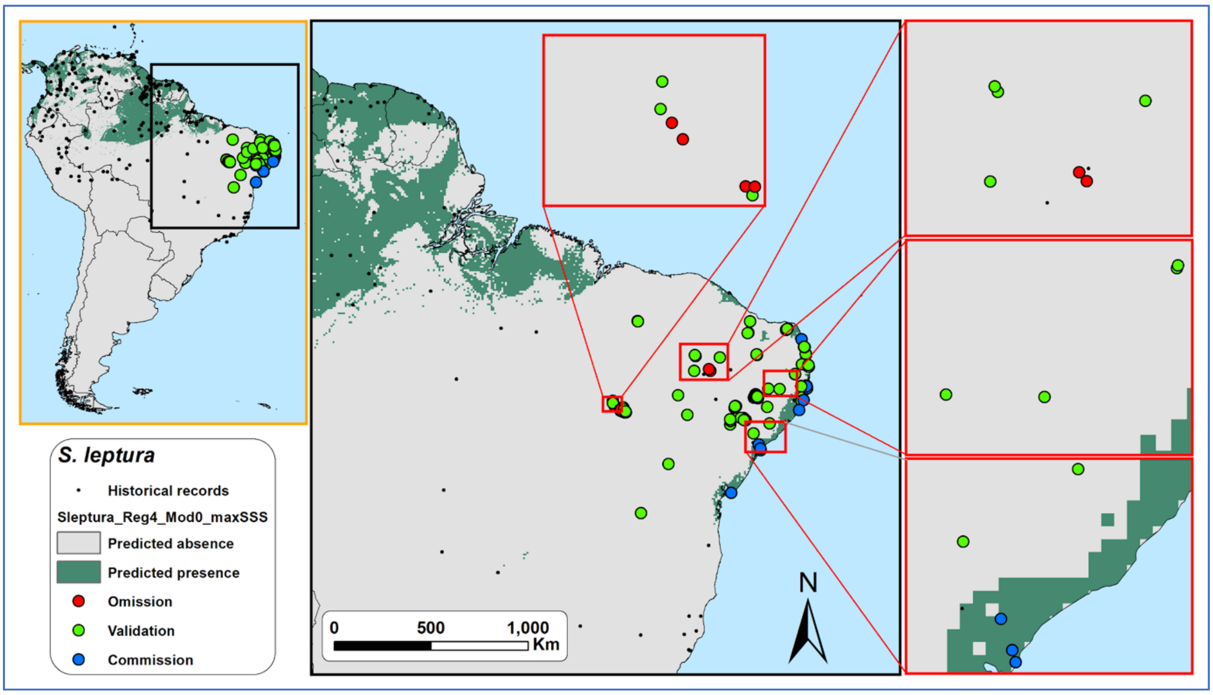

Figure R - Field validation results for the binary maps with the highest accuracy, precision, and specificity scores for *Saccopteryx leptura* in northeastern Brazil. See Fig. A caption for the omission, validation and commission points explanation.

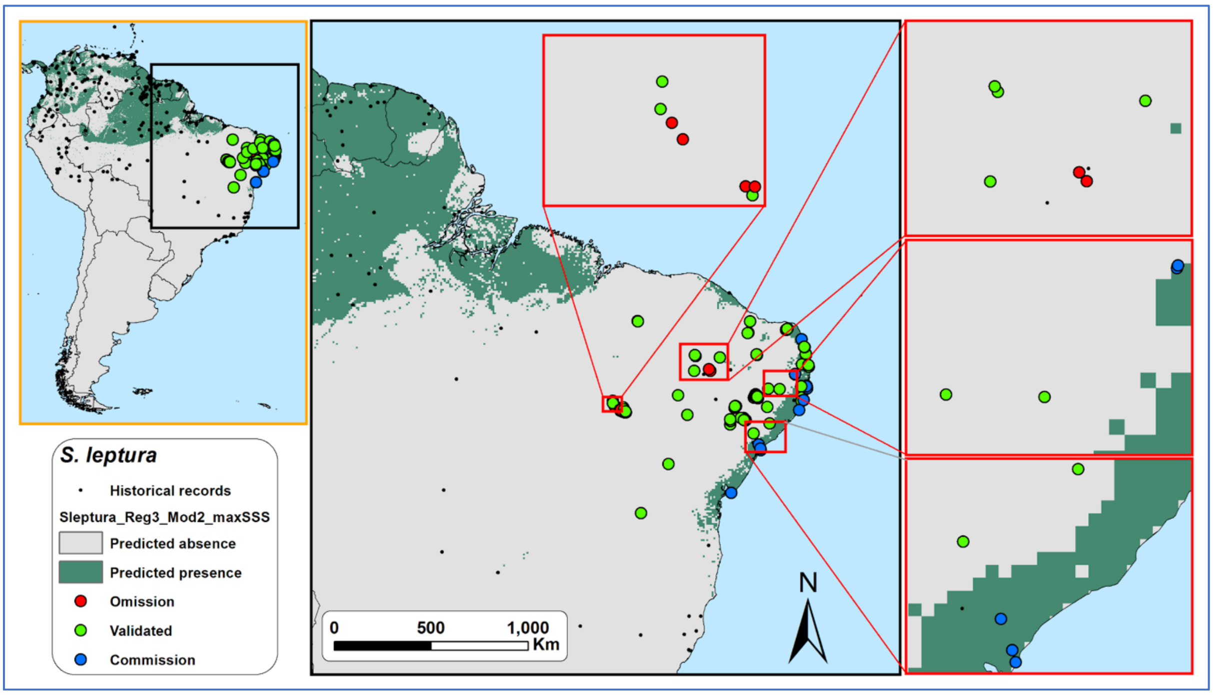

Figure S - Field validation results for the binary maps with the highest f-score score for *Saccopteryx leptura* in northeastern Brazil. See Fig. A caption for the omission, validation and commission points explanation.

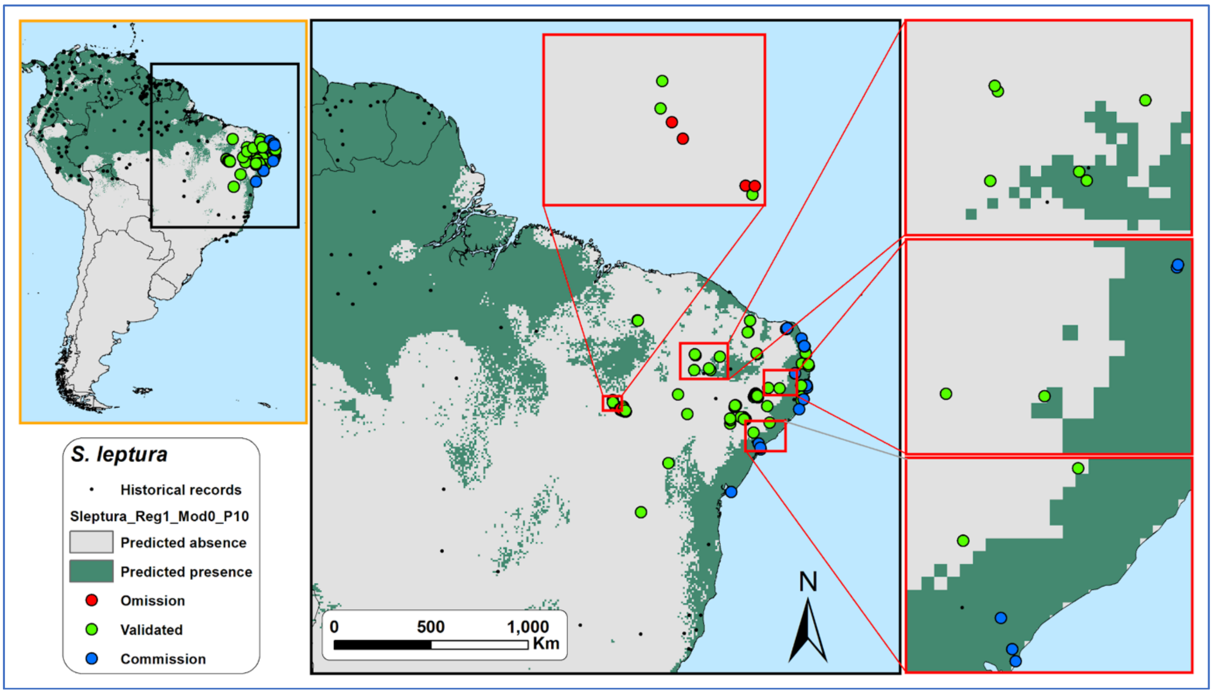

Figure T - Field validation results for the binary maps with the highest g-mean score for *Saccopteryx leptura* in northeastern Brazil. See Fig. A caption for the omission, validation and commission points explanation.

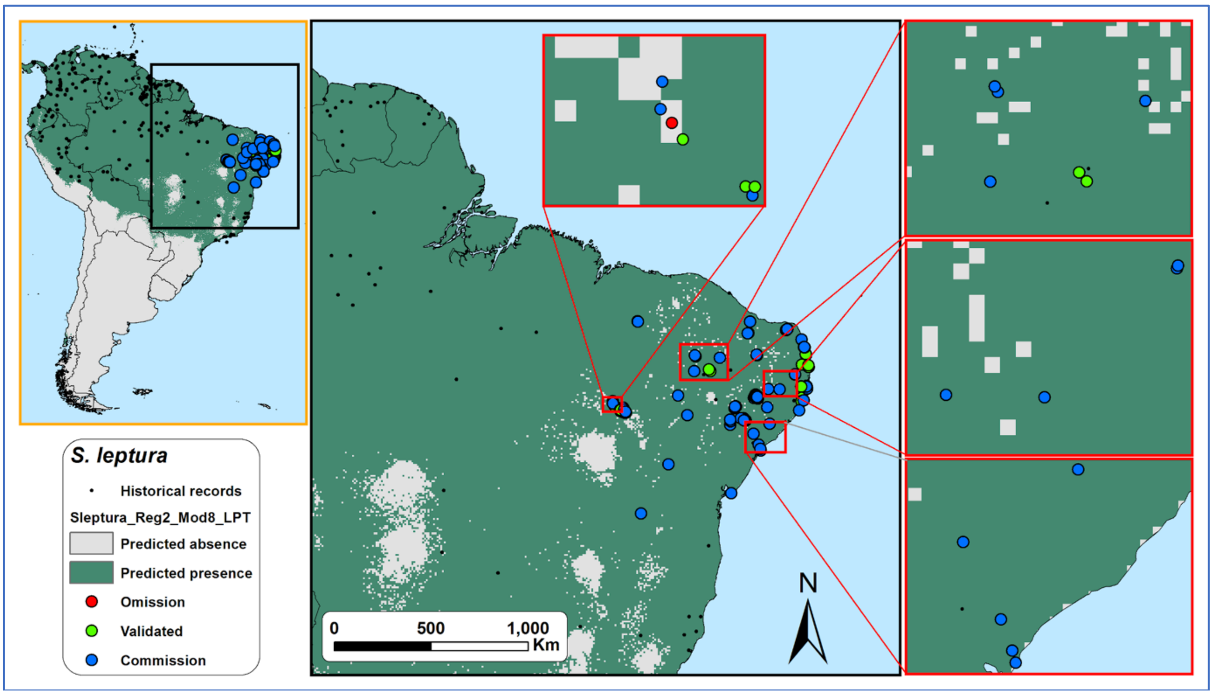

Figure U - Field validation results for the binary maps with the highest sensitivity score for *Saccopteryx leptura* in northeastern Brazil. See Fig. A caption for the omission, validation and commission points explanation.
