# Supplementary material for: Bioacoustics for in situ validation of species distribution modelling: An example with bats in Brazil
Source: PLoS One. 2021 Oct 20;16(10):e0248797. doi: 10.1371/journal.pone.0248797 (PMC8528307; doi:10.1371/journal.pone.0248797)
Supplement: Attachment — Submitted filename: PONE-D-21-07379_reviewer.pdf [file pone.0248797.s007.pdf]

PLOS ONE

Bioacoustics for in situ validation of species distribution modelling: An example with bats in Brazil

--Manuscript Draft--

**Manuscript Number:**	PONE-D-21-07379
**Article Type:**	Research Article
**Full Title:**	Bioacoustics for in situ validation of species distribution modelling: An example with bats in Brazil
**Short Title:**	Using bioacoustics for field validation of bats’ SDMs
**Corresponding Author:**	Frederico Hintze, Ph.D.<br>Universidade Federal de Pernambuco<br>Recife, BRAZIL
**Keywords:**	accuracy; echolocation calls; MaxEnt; predictive distribution maps; thresholds; SDM
**Abstract:**	Species distribution modelling (SDM) gained importance on biodiversity distribution and conservation studies worldwide, including prioritizing areas for public policies and international treaties. Useful for large-scale approaches and estimates, is a plus considering that a minor fraction of the planet is adequately sampled. However, SDM needs to be as reliable as possible. Minimizing errors is challenging, but essential, considering the uses and consequences of such models. In situ validation of the SDM outputs should be a key-step – in some cases, urgent. Bioacoustics can be used to validate and refine those outputs, especially if the focal species' vocalizations are conspicuous and species-specific. This is the case of echolocating bats. Here, we used extensive acoustic monitoring (>120 validation points, covering >758,000 km ^{2}, and >300,000 sound files) to validate MaxEnt outputs for six neotropical bat species in a poorly-sampled region of Brazil. Based on in situ validation, we evaluated four threshold-dependent theoretical evaluation metrics' ability in predicting models' performance. We also assessed the performance of three widely used thresholds to convert continuous SDMs into presence/absence maps. We demonstrated that MaxEnt produces very different outputs, requiring a careful choice on thresholds and modeling parameters. Although all theoretical evaluation metrics studied were positively correlated with accuracy, we empirically demonstrated that metrics based on specificity-sensitivity and sensitivity-precision are better for testing models, considering that most SDMs are based on unbalanced data. Without independent field validation, we found that using an arbitrary threshold for modelling can be a precarious approach with many possible outcomes, even after getting good evaluation scores. Bioacoustics proved to be important for validating SDMs for the six bat species analyzed, allowing a better refinement of SDMs in large and under-sampled regions, with relatively low sampling effort. Regardless of species assessing method used, our research highlighted the vital necessity of in situ validation for SDMs.
**Order of Authors:**	Frederico Hintze, Ph.D.
	Ricardo B. Machado
	Enrico Bernard
**Additional Information:**	
**Question**	**Response**
**Financial Disclosure**<br><br>Enter a financial disclosure statement that describes the sources of funding for the work included in this submission. Review the submission guidelines for detailed requirements. View published research articles from *PLOS ONE* for specific	FH was supported by a PhD grant from the Coordenação de Aperfeiçoamento de Pessoal de Nível Superior–Brasil (CAPES - https://www.gov.br/capes/pt-br) – Finance Code 001<br>EB and RBM have produtivity fellowships conceded by the Conselho Nacional de Desenvolvimento Científico e Tecnológico (CNPq - https://www.gov.br/cnpq/pt-br).<br><br>The funders had no role in study design, data collection and analysis, decision to publish, or preparation of the manuscript.

Powered by Editorial Manager® and ProduXion Manager® from Aries Systems Corporation

examples.<br><br>This statement is required for submission and **will appear in the published article** if the submission is accepted. Please make sure it is accurate.<br><br>**Unfunded studies**<br>Enter: *The author(s) received no specific funding for this work.*<br><br>**Funded studies**<br>Enter a statement with the following details:<br>• Initials of the authors who received each award<br>• Grant numbers awarded to each author<br>• The full name of each funder<br>• URL of each funder website<br>• Did the sponsors or funders play any role in the study design, data collection and analysis, decision to publish, or preparation of the manuscript?<br>• **NO** - Include this sentence at the end of your statement: *The funders had no role in study design, data collection and analysis, decision to publish, or preparation of the manuscript.*<br>• **YES** - Specify the role(s) played.<br><br>* typeset	
**Competing Interests**<br><br>Use the instructions below to enter a competing interest statement for this submission. On behalf of all authors, disclose any competing interests that could be perceived to bias this work—acknowledging all financial support and any other relevant financial or non-financial competing interests.<br><br>This statement is required for submission and **will appear in the published article** if the submission is accepted. Please make sure it is accurate and that any funding sources listed in your Funding Information later in the submission form are also declared in your Financial Disclosure statement.	The authors have declared that no competing interests exist.

View published research articles from *PLOS ONE* for specific examples.<br><br>**NO authors have competing interests**<br><br>Enter: *The authors have declared that no competing interests exist.*<br><br>**Authors with competing interests**<br><br>Enter competing interest details beginning with this statement:<br><br>*I have read the journal's policy and the authors of this manuscript have the following competing interests: [insert competing interests here]*<br><br>* typeset	
**Ethics Statement**<br><br>Enter an ethics statement for this submission. This statement is required if the study involved:<br><br>• Human participants<br>• Human specimens or tissue<br>• Vertebrate animals or cephalopods<br>• Vertebrate embryos or tissues<br>• Field research<br><br>Write "N/A" if the submission does not require an ethics statement.<br><br>General guidance is provided below. Consult the submission guidelines for detailed instructions. **Make sure that all information entered here is included in the Methods section of the manuscript.**	All fieldwork procedures complied with the American Society of Mammalogists´ guidelines for the use of wild mammals in research and education (Sikes et al., 2016) and were previously approved by the Brazilian Ministry of the Environment (SISBIO authorisation n.º 59743-1).

**Format for specific study types**<br><br>**Human Subject Research (involving human participants and/or tissue)**<br>• Give the name of the institutional review board or ethics committee that approved the study<br>• Include the approval number and/or a statement indicating approval of this research<br>• Indicate the form of consent obtained (written/oral) or the reason that consent was not obtained (e.g. the data were analyzed anonymously)<br><br>**Animal Research (involving vertebrate animals, embryos or tissues)**<br>• Provide the name of the Institutional Animal Care and Use Committee (IACUC) or other relevant ethics board that reviewed the study protocol, and indicate whether they approved this research or granted a formal waiver of ethical approval<br>• Include an approval number if one was obtained<br>• If the study involved *non-human primates*, add *additional details* about animal welfare and steps taken to ameliorate suffering<br>• If anesthesia, euthanasia, or any kind of animal sacrifice is part of the study, include briefly which substances and/or methods were applied<br><br>**Field Research**<br><br>Include the following details if this study involves the collection of plant, animal, or other materials from a natural setting:<br>• Field permit number<br>• Name of the institution or relevant body that granted permission	
**Data Availability**<br><br>Authors are required to make all data underlying the findings described fully available, without restriction, and from the time of publication. PLOS allows rare exceptions to address legal and ethical concerns. See the PLOS Data Policy and FAQ for detailed information.	Yes - all data are fully available without restriction

Powered by Editorial Manager® and ProduXion Manager® from Aries Systems Corporation

A Data Availability Statement describing where the data can be found is required at submission. Your answers to this question constitute the Data Availability Statement and **will be published in the article**, if accepted.<br><br>**Important:** Stating 'data available on request from the author' is not sufficient. If your data are only available upon request, select 'No' for the first question and explain your exceptional situation in the text box.<br><br>Do the authors confirm that all data underlying the findings described in their manuscript are fully available without restriction?	
**Describe where the data may be found in full sentences. If you are copying our sample text, replace any instances of XXX with the appropriate details.**<br><br>• If the data are **held or will be held in a public repository**, include URLs, accession numbers or DOIs. If this information will only be available after acceptance, indicate this by ticking the box below. For example: *All XXX files are available from the XXX database (accession number(s) XXX, XXX.).*<br>• If the data are all contained **within the manuscript and/or Supporting Information files**, enter the following: *All relevant data are within the manuscript and its Supporting Information files.*<br>• If neither of these applies but you are able to provide **details of access elsewhere**, with or without limitations, please do so. For example:<br><br>*Data cannot be shared publicly because of [XXX]. Data are available from the XXX Institutional Data Access / Ethics Committee (contact via XXX) for researchers who meet the criteria for access to confidential data.*<br><br>*The data underlying the results presented in the study are available from (include the name of the third party*	All relevant data are within the manuscript and its Supporting Information files.

and contact information or URL).<br>• This text is appropriate if the data are owned by a third party and authors do not have permission to share the data.<br><br>* typeset	
Additional data availability information:	

Powered by Editorial Manager® and ProduXion Manager® from Aries Systems Corporation

Manuscript Click here to access/download;Manuscript;Manuscript.docx

Bioacoustics for *in situ* validation of species distribution modelling: An example with bats in Brazil

Using bioacoustics for field validation of bats' SDMs

Frederico Hintze [a,b], Ricardo B. Machado [c], Enrico Bernard [a]

[a] Laboratório de Ciência Aplicada à Conservação da Biodiversidade, Departamento de Zoologia, Universidade Federal de Pernambuco, Rua Professor Nelson Chaves s/n, Cidade Universitária, Recife PE 50670-420, Brasil.

[b] Programa de Pós-graduação em Biologia Animal, Departamento de Zoologia, Universidade Federal de Pernambuco, Av. Prof. Moraes Rego, 1235, Recife PE 50670-901, Brasil.

[c] Departamento de Zoologia, Instituto de Ciências Biológicas, Campus Darcy Ribeiro, Universidade de Brasília, Brasília DF 70910-900, Brasil

Abstract

Species distribution modelling (SDM) gained importance on biodiversity distribution and conservation studies worldwide, including prioritizing areas for public policies and international treaties. Useful for large-scale approaches and estimates, is a plus considering that a minor fraction of the planet is adequately sampled. However, SDM needs to be as reliable as possible. Minimizing errors is challenging, but essential, considering the uses and consequences of such models. *In situ* validation of the SDM outputs should be a key-step – in some cases, urgent. Bioacoustics can be used to validate and refine those outputs, especially if the focal species' vocalizations are conspicuous and species-specific. This is the case of echolocating bats. Here, we used extensive acoustic monitoring (>120 validation points, covering >758,000 km^2, and >300,000 sound files) to validate MaxEnt outputs for six neotropical bat species in a poorly-sampled region of Brazil. Based on *in situ* validation, we evaluated four threshold-dependent theoretical evaluation metrics' ability in predicting models' performance. We also assessed the performance of three widely used thresholds to convert continuous SDMs into presence/absence maps. We demonstrated that MaxEnt produces very different outputs, requiring a careful choice on thresholds and modeling parameters. Although all theoretical evaluation metrics studied were positively correlated with accuracy, we empirically demonstrated that metrics based on specificity-sensitivity and sensitivity-precision are better for testing models, considering that most SDMs are based on unbalanced data. Without independent field validation, we found that using an arbitrary threshold for modelling can be a precarious approach with many possible outcomes, even after getting good evaluation scores. Bioacoustics proved to be important for validating SDMs for the six bat species analyzed, allowing a better refinement of SDMs in large and under-sampled regions, with relatively low sampling effort. Regardless of species assessing method used, our research highlighted the vital necessity of *in situ* validation for SDMs.

Keywords: accuracy, echolocation calls, MaxEnt, predictive distribution maps, thresholds, SDM.

Introduction

Species distribution modelling (SDM) gained importance in the development of studies on biodiversity distribution and conservation worldwide [1, 2]. These distribution models can be produced and refined by crossing species presence records with biological and non-biological variables and environmental data [e.g., 3]. Such modelling can be beneficial for large-scale approaches and estimates, a plus considering that fewer parts of planet Earth were adequately sampled [4, 5]. Moreover, SDM has been used to support decision-making processes, including prioritizing areas and regions in public policies and international treaties [2, 5].

For biodiversity conservation purposes, SDM should be as reliable as possible. For instance, imprecise models can undermine the calculation/estimate of a species' occupancy, a criterion used to assess its conservation status, for example [6]. Depending on the quality of the input data and modelling parameters chosen, the predictions created may not translate precisely the species' distribution [7, 8]. Known as commission (false positives) and omission (false negatives) errors, they can inflate or reduce a given *taxon*'s potential distribution. Minimizing such errors is a challenge for the spatial modelling science [4, 7, 9], but essential considering the uses and consequences such models may have [10]. Therefore, *in situ* validation of the SDM outputs should be a critical step – in some cases, urgent [10, 11].

Certifying that a species is present in a given area is not always straightforward, and different and innovative approaches have been proposed for such task [e.g., e-DNA [12] or satellite images [13]]. Bioacoustics is one of such techniques and has been used for a long time to record species presence/absence for birds, cetaceans, and amphibians [14]. Bats are a widespread group, using many habitats and resources, and most of the 1400 known species

depend on echolocation for navigation and food acquisition [15]. The bats' echolocation system is based on ultrasonic signals which, although above the human hearing capacity, thanks to recent electronics advances, can be easily recorded [16]. Moreover, most bats have conspicuous and species-specific calls, allowing precise identification of the emitter's identity and making the record of its presence accurate [16]. Therefore, bioacoustics can be a useful technique applied to the *in situ* validation and refinement of SDM outputs for echolocating bats.

Here, we used extensive *in situ* monitoring (> 120 validation points, covering > 758,000 km^2, and > 300,000 sound files) of echolocation calls to validate the outputs of the most used SDM algorithm [MaxEnt; 3] for six neotropical bat species in a poorly-sampled part of Brazil. Using independent acoustic data collected, we (a) evaluated the ability of four threshold-dependent theoretical evaluation metrics in predicting models' performance, and (b) assessed the ability of three widely used thresholds to convert continuous species habitat suitability models into binary (presence/absence) maps.

Materials and Methods

Historical species records

In our analysis, we selected six neotropical bat species whose echolocation calls are well-known, species-specific and unequivocally identifiable: *Noctilio leporinus*, *Promops centralis*, *Promops nasutus*, *Pteronotus gymnonotus*, *Pteronotus personatus*, and *Saccopteryx leptura* [17-19]. We gathered distribution records for these species from a bibliographic revision using the following online databases and search engines: the Vertebrate Zoology Database of the American Museum of Natural History (http://sci-web-001.amnh.org/db/emuwebamnh/index.php); the database of the Division of Mammals Collections of the Smithsonian National Museum of Natural History (https://collections.nmnh.si.edu/search/mammals/); the Global Biodiversity Information

Facility (www.gbif.org); the SpeciesLink network (http://www.splink.org.br); Google Scholar (scholar.google.com); the Web of Science (www.webofknowledge.com); Scopus (www.scopus.com); the Periódicos CAPES (www.periodicos.capes.gov.br); and the Scientific Electronic Library Online (www.scielo.br). We also reviewed occurrences in studies available online such as Barquez, Ojeda [20] and Gardner [21]. Each record was checked for duplication of localities and, eventually, to correct location or taxonomy problems [22]. For example, following Gardner [21], we treated *P. nasutus* as a monotypic species (considering *Promops nasutus ancilla*, *P. nasutus pamana*, *P. nasutus fosteri* or *P. nasutus downsi* as simply *Promops nasutus*). Overall, we gathered a total of 1277 single records for the six studied bat species: 590 records for *Noctilio leporinus*, 70 for *Promops centralis*, 71 for *Promops nasutus*, 120 for *Pteronotus gymnonotus*, 95 for *Pteronotus personatus*, and 331 for *Saccopteryx leptura* (Fig 1, and S1 Table in supplementary material).

Fig 1. Historical distribution records assembled from the literature review and the 129 acoustic sampling points used for the validation of SDM of six neotropical bat species (*Noctilio leporinus*, *Promops centralis*, *Promops nasutus*, *Pteronutus gymnonotus*, *Pteronotus personatus*, and *Saccopteryx leptura*).

Distribution modelling procedure

We used SDMtoolbox 2.4 for ArcGIS [23] to create an environmental heterogeneity map with the bioclimatic variables of Worldclim [24]. To reduce the potential bias caused by autocorrelation, we then used the Spatial Rarefy Occurrence Data tool of the SDMtoolbox 2.4 package to delete records under the same environmental conditions within 25 km from each other [25]. Other studies usually use a 10km distance between points [e.g. 26, 27, 28], but given the topographic and environmental heterogeneity of the studied region, we chose 25 km as a spatial filter to avoid eventual spatial bias on historical records [29]. This process ensured that all historical records gathered in our revision corresponded to a unique spatial sample, reducing occurrence data from 1277 to 899 single localities: 375 localities for *Noctilio leporinus*, 61 for

Promops centralis, 65 for *Promops nasutus*, 87 for *Pteronotus gymnonotus*, 66 for *Pteronotus personatus*, and 245 for *Saccopteryx leptura*.

We used MaxEnt 3.4 [3] to generate potential species distribution models for the six selected species based on a set of variables at a 5 km^2 resolution. We used the 19 bioclimatic variables plus elevation available at the Worldclim data website [24] and Globcover 2009 [30] as a categorical variable for land cover. To reduce the multicollinearity among the predictor variables, we performed a preliminary model with all variables and checked the weight of each according to their contributions, using Jackknife tests. Next, using the Correlations and Summary Stats tool of the SDMtoolbox 2.4 package, we obtained the correlation and covariances matrices and removed highly correlated variables (i.e., those with the lowest value when the pairwise correlation was > 0.7) [31]. Therefore, we used different variables for each modelled species (S2 Table, in supplementary material).

We used a logistic output to produce our models and obtain continuous suitability values for species habitat suitability, which varies from 0 (lowest suitability) to 1 (highest suitability) [3, 32]. Since default regularization values lead to overfitted models when using spatial filtering [28], we calibrated the models with different regularization multiplier values (default 1.0, 2.0, 3.0, and 4.0) [28, 33]. To generate overall predictive distribution models, we used 75% of the data for calibration and 25% for internal evaluation (testing data).

We used ten cross-validation replications to test all models and calculate confidence intervals, resulting in 40 models for each species, and a total of 240 continuous suitability models for all species. Since we aimed to use bioacoustics data to validate binary maps (presence-absence), we used three widely used thresholds to convert the continuous suitability values of each model into binary maps: lowest presence threshold (hereafter LPT) [27]; 10th percentile of the predicted values (hereafter P10) [27]; and maximum sum of sensitivity and specificity (hereafter maxSSS) [34, 35]. LPT is considered the least conservative threshold,

whereas maxSSS the most [34, 36]. We produced a total of 720 binary models (i.e., 120 binary models for each species: 6 species x 120 binary models).

Using MaxEnt's background and sample predictions for each model, we then evaluated each 120 species binary models for their predicting performance using four threshold-dependent theoretical evaluation metrics: (1) overall accuracy (hereafter OAcc) [37, 38]; (2) Cohen's maximized kappa statistics (hereafter P-kappa) [39, 40]; (3) True Skill Statistics (hereafter TSS) [38, 41]; and, (4) Symmetric Extremal Dependence Index (hereafter SEDI) [42]. OAcc measures the model predicted accuracy using the rate of correct classifications (true positive + true negative) and ranges from 0 to 1; while P-kappa, TSS and SEDI measures the model predicted accuracy taking into account the predicted accuracy of a by-chance model [38, 42]. Pkappa, TSS and SEDI range from -1 to +1, where values ≤ 0 suggest a performance equal or worse than random, and as close the values get to +1, the better the prediction [38, 42]. For the math behind each theoretical evaluation metrics calculation, please consult Allouche, Tsoar [38], Cohen [39], Peirce [41], and Wunderlich, Lin [42].

As expected, the theoretical metrics did not converged on the same best models, therefore we selected the two best-scored binary models based on each metric (i.e., the two best-scored based on OAcc, the two best-scored based on P-kappa, and so on…) for each of the thresholds used (LPT, P10, and maxSSS). Then, we did the field validation on the resulting 24 theoretical best binary models for each species, 144 binary models in total of the six studied species.

Field validation sampling and acoustic identification

For the selection of the sampling points for field validation, we summed the average potential distribution outputs of the six species to identify regions with highest and lowest suitability of species occurrence, but without historical records. Considering the potential

distribution of those species covered extensive areas, we focused our field validation on 129 randomly-selected sampling points along 1000 km from east to west and 1000 km from north to south, covering an area of 758,193 km^2, in the Northeastern part of Brazil (Fig 1, and S3 Table supplementary material).

Between March 2014 and January 202[illegible] we employed passive acoustic monitoring to sample bat echolocation calls in the 129 sampling points (Fig 1, and S3 Table in supplementary material), using a combination of SM2Bat+, SM3BAT, and SM4BAT-FS ultrasound recorders (Wildlife Acoustics Inc., Massachusetts, USA). Since the highest frequency used by the studied species is ~60 kHz (*Noctilio leporinus*) [18], we configured the bat detectors with a minimum sampling rate of 384 kHz, enough to detect and record our focal species without distortions (e.g., aliasing). Each sampling point was acoustically monitored for at least two nights, recording continuously from 30 minutes before sunset until 30 minutes after sunrise. Since bat activity and the reception of the calls can be affected by weather and local conditions, we sampled only during nights with temperature > 15ºC, without strong winds (< 5 m/s) or rain [16, 43, 44]. We set the microphones at 45° to the ground, avoiding highly cluttered areas [16, 43, 44].

We used Raven Pro 1.5 (The Cornell Lab of Ornithology 2014) for the acoustic analysis performed in the laboratory. We configured the spectrograms to DFT equals 1024, 96% overlap, window length to 1 ms, using Hamming windows. We only analyzed sequences containing a minimum of three search calls with a good signal-to-noise ratio (> 15 dB) [45, 46]. We performed manual acoustic identification using qualitative (e.g., call structure and modulation) and quantitative parameters (e.g., frequency of maximum energy, maximum and minimum frequency, call duration, etc.), following previously published studies on neotropical bat acoustic identification [e.g., 17-19, 46, 47, 48]. We did not use feeding-buzzes (and calls immediately before and after) or social-calls for identification purposes. Although the chosen

species have different natural histories, all are considered as common in the sampled region and can be easily detected and identified by acoustics. All fieldwork procedures complied with the American Society of Mammalogists´ guidelines for the use of wild mammals in research and education [49] and were previously approved by the Brazilian Ministry of the Environment (SISBIO n.º 59743-1).

Models' field validation

We evaluated the total 144 selected binary models for the six focal species against the results from the acoustic monitoring performed in the field. We used a confusion matrix to compare the accuracy of the binary maps, where the observed presence and absence cases from the acoustic monitoring were compared against the predicted presence and absence of the models. This procedure allowed us to quantify true positives (TP), true negatives (TN), false negatives (FN; omission errors, type I errors), and false positives (FP; commission errors, type II errors).

We used six metrics for the model performance evaluation: (1) Accuracy, to quantify how often the model is correct in the overall prediction [Accuracy = (TP + TN) / total cases]; (2) Precision, to quantify how often is the model correct when it predicts the occurrence of the species [Precision = TP / (TP + FP)]; (3) Sensitivity (true positive rate, or recall), to quantify the ability of the model to predict species occurrence [Sensitivity = TP / (TP + FN)]; (4) Specificity (or true negative rate), which quantifies the ability of the model to predict species absence [Specificity = TN / (FP + TN)]; (5) geometric mean of sensitivity and specificity (g-mean), is a performance metric for imbalanced classifications, high g-mean indicates a right balance between sensitivity and specificity [g-mean = √ (Sensitivity . Specificity)]. If the species presence classification performance is weak, the g-mean will be low even with an excellent species absence classification performance [50]; and (6) harmonic mean of precision

and sensitivity [f-score = $(2 \cdot \text{Precision} \cdot \text{Sensitivity}) / (\text{Precision} + \text{Sensitivity})$], gives the same importance to precision and sensitivity, i.e., high F-score indicates excellent model performance on the minority class [50, 51]. The commission error rate is inversely proportional to sensitivity (= 1 - sensitivity), whereas the omission error rate is inversely proportional to specificity (= 1 - specificity).

To assess the overall performance of the theoretical evaluation metrics (OAcc, P-kappa, TSS, and SEDI), we performed a Spearman rank-order correlation between those scores and the post-validation performance metrics scores (accuracy, precision, sensitivity, specificity, g-mean, and f-score). To test differences in the prediction performances (using the post-validation performance metrics scores) between the thresholds used (LPT, maxSSS, and P10), we employed the Kruskal-Wallis test with Mann-Whitney pairwise *post hoc* test. The thresholds were tested with both all species together and separately for each one.

Result

SDM's and acoustic field validation

The acoustics sampling performed in this study resulted in more than 1.5 TB of raw sound files, where more than 300,000 sound files contained bat calls. We identified echolocation calls of *Noctilio leporinus* in 38 points (29,4%), of *Promops centralis* in 23 (17,8%), *Promops nasutus* in 44 (34,1%), *Pteronotus gymnonotus* in 53 (41,1%), *Pteronotus personatus* in 21 (16,3%), and *Saccopteryx leptura* in 24 of the 129 sampled points (18,6%) (S3 Table, in supplementary material).

After field validation, the performance scores varied considerably between the 144 binary models: accuracy varied from 0.16 to 0.81, precision varied between 0.09 and 0.59, sensitivity varied from 0.17 to 1, specificity from 0 to 0.86, g-mean from 0 to 0.75 and f-score from 0.12 to 0.60 (S4 Table, in supplementary material). We registered the highest accuracy score (0.81) in a maxSSS thresholded map of *Saccopteryx leptura*, the highest precision score

(0.59) in a maxSSS thresholded map of *Noctilio leporinus*, and the highest sensitivity score (= 1) in LPT thresholded maps of four species (*Noctilio leporinus*, *Pteronotus gymnonotus*, *Promops nasutus*, and *Pteronotus personatus*) (S4 Table and S5 Maps, in supplementary material). The highest specificity score (0.86) was recorded in maxSSS thresholded maps of two species (*Noctilio leporinus* and *Saccopteryx leptura*), the highest g-mean score (0.75) in a P10 thresholded map of *Saccopteryx leptura*, and the highest f-score score (0.60) in a maxSSS thresholded maps of *Pteronotus gymononotus* (S4 Table and S5 Maps, in supplementary material).

Model evaluation vs. field validation

All theoretical model evaluation metrics analysed exhibited a significant monotonic positive correlation with accuracy and specificity (Table 1). However, the evaluation metrics exhibited an overall significant negative correlation with sensitivity (Table 1). We also found very weak monotonic correlations between the evaluation metrics and precision (Table 1). Although negative, the correlation between the theoretical evaluation metrics and f-score was not significant (Table 1). Only TSS and overall accuracy exhibited significant monotonic positive correlations with g-mean (Table 1).

Table 1. Spearman rank-order correlation results of the theoretical evaluation metrics and the post-validation performance metric scores for the distribution modelling of six neotropical bats based on bioacoustics field validation in northeastern Brazil.

Theoretical evaluation metric	Validation performance metric	rs	n	p (w/ Bonferroni correction)
TSS	Accuracy	0.36	144	<0.001
	Precision	0.08	144	1
	Sensitivity	-0.54	144	<0.001
	Specificity	0.47	144	<0.001
	G-mean	0.43	144	<0.001
	F-score	-0.09	144	1
OAcc	Accuracy	0.56	144	<0.001

	**Precision**	0.15	144	1
	**Sensitivity**	-0.86	144	<0.001
	**Specificity**	0.74	144	<0.001
	**G-mean**	0.57	144	<0.001
	**F-score**	-0.19	144	0.91
**P-kappa**	**Accuracy**	0.27	144	<0.05
	**Precision**	0.06	144	1
	**Sensitivity**	-0.37	144	<0.001
	**Specificity**	0.35	144	<0.001
	**G-mean**	0.38	144	<0.001
	**F-score**	-0.02	144	1
**SEDI**	**Accuracy**	0.26	97	0.31
	**Precision**	0.00	97	1.00
	**Sensitivity**	-0.38	97	<0.05
	**Specificity**	0.35	97	<0.05
	**G-mean**	0.29	97	0.18
	**F-score**	-0.18	97	1

rs, Spearman correlation coefficient; n, number of pairwise comparisons.

Thresholds vs. validation

Based on the output maps of all species together, LPT threshold maps exhibited significantly the lowest overall average accuracy, precision, specificity, and g-mean scores of the three thresholds tested (Figs 2-8, and S6 Table in supplementary material). While P10 exhibited a significantly higher average f-score than maxSSS, we found no significant differences between the f-score results between P10 and LPT, and between maxSSS and LPT (Fig 2, and S6 Table in supplementary material). P10 obtained overall average sensitivity and specificity scores between the other two thresholds (Fig 2, and S6 Table in supplementary material). LPT maps scored the highest average sensitivity but also presented low average specificity scores. Note that while LPT's sensitivity scores near one, its specificity scores are also near zero (Fig 2). We found this same LPT and maxSSS behavior when we analyzed all species separately (Figs 3-8, and S6 Table in supplementary material). One LPT map for *N. leporinus* (Fig 9) exemplifies this odd behavior, where omission errors are minimal, but

commission errors are maximum (sensitivity = 1, specificity = 0, and g-mean = 0). Even the LPT map with the highest g-mean score (*P. nasutus*, Fig 10) represented a commission error rate of ~81% and an omission error rate of ~7% (sensitivity = 0.93, specificity = 0.19, and g-mean = 0.42). While maxSSS scored the highest overall average accuracy and specificity, its maps also exhibited the lowest average sensitivity scores (S6 Table in supplementary material). Nevertheless, in contrast to LPT, maxSSS' sensitivity and specificity average scores are similar, thus presenting higher g-mean scores than LPT (Fig 2, and S6 Table in supplementary material). This was clear when the maxSSS with the highest g-mean (Fig 11), represented an omission error of ~29% and a commission error of ~25% (sensitivity = 0.71, specificity = 0.75, and g-mean = 0.73). All thresholds significantly presented different specificity and specificity scores, but we found no significant differences between the overall accuracy, precision, and g-mean scores of maxSSS and P10 threshold maps (Fig 2, and S6 Table in supplementary material).

Fig 2. Box-plots of the accuracy, precision, sensitivity, specificity, g-mean, and f-score scores of LPT, maxSSS, and P10 thresholded maps.
Lower and top box boundaries represent the 25th and 75th percentile, respectively. The point inside the box represents the average, while the line represents the median. Error lines represent 1.5*interquartile range, and any asterisk outside the error lines represent outliers. Different letters indicate significant differences between groups ($p < 0.05$).

Fig 3. Box-plots of the accuracy, precision, sensitivity, specificity, g-mean, and f-score scores of *Noctilio leporinus*' LPT, maxSSS, and P10 thresholded maps.
See Fig 2 caption for box-plot explanation. Different letters indicate significant differences between groups ($p < 0.05$).

Fig 4. Box-plots of the accuracy, precision, sensitivity, specificity, g-mean, and f-score scores of *Promops centralis*' LPT, maxSSS, and P10 thresholded maps.
See Fig 2 caption for box-plot explanation. Different letters indicate significant differences between groups ($p < 0.05$).

Fig 5. Box-plots of the accuracy, precision, sensitivity, specificity, g-mean, and f-score scores of *Pteronotus gymnonotus*' LPT, maxSSS, and P10 thresholded maps.
See Fig 2 caption for box-plot explanation. Different letters indicate significant differences between groups ($p < 0.05$).

Fig 6. Box-plots of the accuracy, precision, sensitivity, specificity, g-mean, and f-score scores of *Promops nasutus*' LPT, maxSSS, and P10 thresholded maps.
See Fig 2 caption for box-plot explanation. Different letters indicate significant differences between groups ($p < 0.05$).

Fig 7. Box-plots of the accuracy, precision, sensitivity, specificity, g-mean, and f-score scores of *Pteronotus personatus*' LPT, maxSSS, and P10 thresholded maps.
See Fig 2 caption for box-plot explanation. Different letters indicate significant differences between groups ($p < 0.05$).

Fig 8. Box-plots of the accuracy, precision, sensitivity, specificity, g-mean, and f-score scores of *Saccopteryx leptura*' LPT, maxSSS, and P10 thresholded maps.
See Fig 2 caption for box-plot explanation. Different letters indicate significant differences between groups ($p < 0.05$).

Fig 9. Field validation results for the LPT binary map with the highest accuracy score (= 1) for *Noctilio leporinus* in northeastern Brazil.
Omission errors are minimal but commission errors are maximum (sensitivity = 1, specificity = 0, and g-mean = 0). 'Omission' points represent locations where the model did not predict the species occurrence, but the species was detected during the acoustics monitoring; 'Validated' points represent locations where the model predict the species occurrence and the species was detected during the acoustics monitoring or locations where the model did not predict the species occurrence, and the species was detected during the acoustics monitoring; 'commission' points represent locations where the model predict the species occurrence but the species was not detected during the acoustics monitoring.

Fig 10. Field validation results for the LPT binary map with the highest g-mean score (= 0.42) for *Promops nasutus* in northeastern Brazil.
Omission errors are low but commission errors are very high (sensitivity = 0.93, specificity = 0.19). See Fig 7 caption for the explanation on omission, validation and commission points.

Fig 11. Field validation results for the maxSSS binary map with the highest g-mean score (= 0.73) for *Saccopteryx leptura* in northeastern Brazil.
Omission errors and commission errors are balanced (sensitivity = 0.71, specificity = 0.75). See Fig 7 caption for the explanation on omission, validation and commission points.

Analyzing species by species, LPT maps once again significantly exhibited the highest average sensitivity scores. However, it also showed significantly the lowest average accuracy, specificity, and g-mean scores for all six species studied (Figs 3-6, and S6 Table in supplementary material). In the cases of *N. leporinus* and *S. leptura* (Figs 3 and 8), LPT maps also significantly exhibited the lowest average precision and f-score, and the lowest average precision scores for *P. nasutus* and *P. personatus* among the three thresholds tested (Figs 6 and 7, and S6 Table in supplementary material). Thresholded maps based on maxSSS significantly exhibited the lowest average sensitivity scores among the three thresholds tested for *N. leporinus* and *P. gymnonotus* (Figs 3 and 5, and S6 Table in supplementary material). Still, maxSSS also showed significantly the highest average specificity and g-mean scores for the

same species. The maxSSS maps also presented the highest average accuracy for *N. leporinus* and *P. personatus*, and the highest average g-mean for *S. leptura* among the three thresholds tested (Figs 3, 7 and 8, and S6 Table in supplementary material). *Saccopteryx leptura*'s P10 maps significantly exhibited the highest average g-mean of the three thresholds tested (Fig 8, and S6 Table in supplementary material). We found no differences between the performance scores based on maxSSS and P10 in all *P. centralis* and *P. nasutus* maps (Figs 4 and 6). We also found no differences between the three thresholds in precision and f-score for *P. centralis* and *P. personatus* (Figs 4 and 7), f-score of *P. nasutus* (Fig 6), and precision scores of *P. gymnonotus* maps (Fig 5). See S5 Maps in supplementary material for the best performing binary maps for the six species.

Discussion

This study evaluated and validated species binary distribution models using a combination of acoustic data collected in the field and simple performance metrics. Bioacoustics proved to be a very effective method for the *in situ* validation of SDM for six neotropical bat species in a large and poorly-sampled area in Brazil. For species with conspicuous vocalizations – like bats – this method has the potential to better refine SDMs in large and under-sampled regions, requiring relatively low sampling effort. This is quite useful in tropical areas, usually bat species-rich, but frequently understudied [52, 53]. However, we also demonstrated that a careful decision on the modelling parameters and thresholds used is pivotal since, depending on the combination, they can produce very different outputs. Our observations highlight the importance and necessity of *in situ* validation of SDM’ outputs.

Field validation of SDM is unusual [e.g., 54, 55], and very rare for bats [e.g., 10, 56, 57]. Still, we empirically demonstrated that independent field surveys are the best approach to corroborate the predictions made by modelling, especially in subsampled regions with high biodiversity like the Neotropics. We are aware that *in situ* validation of SDM is not always

possible, as that will depend on the focal species, its extension of occurrence, survey methods, and the type and accessibility of the potential area modelled. However, field validation of SDM – in smaller or focally-selected parts of the predicted distribution, or randomly-selected regions – should be imperative. This is especially important in a conservation-focused scenario dealing with such high habitat changes due to anthropogenic causes. Modelling species distributions without proper *in situ* validation may result in inaccurate outputs, compromising the implementation of better conservation policies or species management plans, for example [5, 9, 57, 58]. This can be particularly serious in the case of models with actual low sensibility (high omission errors).

Theoretical model evaluation metrics and thresholds vs. validation

We found that all theoretical model evaluation metrics studied here correlated positively with accuracy. However, caution is necessary, since the most detected species (*P. gymnonotus*) was recorded in only 41% of the sampled points (meaning an unbalanced class data, i.e., in this case absences are higher than presences). In situations like this, any random model predicting more absences than presences would be benefited by an evaluation metric that does not take into account results by chance – this is the case of accuracy [59]. Therefore, accuracy should not be used when data used to train and/or test the models is unbalanced. Here, we empirically demonstrated that sensitivity-specificity and precision-sensitivity metrics, as g-mean and f-score, are better performance measures for the SDMs evaluation than accuracy [50, 59]. For example, the *Pteronotus gymnonotus'* distribution output with the highest accuracy also had the third-highest omission rate. In opposition, the model with the highest g-mean and f-score also presented low omission scores. These results were not a threshold-related since it occurred in two different maxSSS models, evidencing the unbalanced nature of our SDM outputs and the problem of using accuracy to measure model performance. Considering the

majority of SDMs are based on unbalanced data, instead of accuracy, the use of sensitivity-specificity and precision-sensitivity metrics should be mandatory to test the models as they are not affected by unbalanced class data sets [50, 59].

Surprisingly, we also found that all theoretical threshold-dependent evaluation metrics tested here exhibited an overall significant negative correlation with sensitivity and a significant positive correlation with specificity. This means that models with higher evaluation scores predicted better locations with actual species absence than species presence. West, Kumar [60] reported similar findings after field validation of MaxEnt's invasive cheatgrass species models. This is probably because bioclimatic variables' values are more homogeneous in species presence locations than in absence. Nevertheless, this 'issue' will be less a concern if the modeler's goal is to balance actual presences and absences, as we found positive correlations between evaluation metrics and g-mean or f-score.

We found that threshold performances varied largely. Despite having almost no omission errors, the LPT models exhibited higher commission error rates and lower accuracy, g-mean and precision scores. Thus, at least for the six widespread neotropical bat species studied here, we were able to empirically confirm Liu, White [35]’s findings: maps based on the LPT threshold are unsuitable for species distribution modelling. In our study, LPT-thresholded maps of the two species with the most historical records (*N. leporinus* and *S. leptura*) also had a worse performance than the other four LPT-modeled scores. Although widespread and common, *N. leporinus* is a piscivorous bat species, strongly related to water bodies, and *S. leptura* is a forest-dwelling species that forage next to edges [61, 62]. Thus, these two species as less generalist than the other four we analyzed and the use of a less conservative threshold can be detrimental in those cases. Therefore, knowledge of the species' natural history and the use of land cover data in the models might be fundamental for best SDM practices and the better output results [63].

But contrary to Liu, White [35], we also found that some maxSSS maps with high accuracy scores were highly overfitted (exhibiting low sensitivity/high omission rates), sometimes excluding historical locations for some species. Nevertheless, we also found that some maxSSS and P10 thresholded maps performed reasonably well, exhibiting balanced results between sensitivity and specificity (displaying high g-mean scores). In general, even showing higher omission errors than LPT, P10 thresholded models performed best in predicting actual species occurrences, while maxSSS models performed best in predicting where we did not record the species in the field. Several authors agree that threshold selection (as other parameters) has a high impact on the binary map (presence/absence) outputs and the models' predictive capacity [e.g., 6, 8]. Several thresholds have been proposed and evaluated; however, most of those evaluations are based on theoretical evaluation metrics without independent field validation data. After using *in situ* validation in our study, we are cautionary about some studies still proposing to model species distribution using a single threshold. Without independent field validation of the results, we found that using an arbitrary threshold for modelling can be a precarious approach with many possible outcomes, even if getting good evaluation scores. Undoubtedly, validating the models using part of the historical occurrence points is faster and less laborious than using independent data collected in the field. However, one cannot guarantee if the species are still present in historical points in databases such as GBIF [64]. Hence, independent field data are the safest way to validate the species´ presence in the modelled region.

Authors' contributions

Frederico Hintze: Conceptualization, Methodology, Validation, Formal analysis, Investigation, Data Curation, Writing - Original Draft, Writing - Review & Editing, Project administration; **Ricardo B. Machado:** Conceptualization, Methodology, Formal analysis, Data curation, Writing - Review & Editing; **Enrico Bernard:** Conceptualization, Resources,

Writing - Original Draft, Writing - Review & Editing, Supervision, Project administration, Funding acquisition.

Acknowledgments

We thank all those who helped during fieldwork. We are also grateful to ICMBio and the surveyed conservation areas' staff for all the support. FH thanks the Raven Team Bioacoustics Research Program for the free copy of Raven software. This manuscript was based in one of the chapters of FH's PhD Thesis at PPGBA/UFPE, and we thank to the five doctoral examiners for their constructive comments during the defense. This study was financed in part by the Coordenação de Aperfeiçoamento de Pessoal de Nível Superior–Brasil (CAPES) –Finance Code 001 supporting FH with a PhD grant. EB and RBM have CNPq fellowships.

References

1. Zimmermann NE, Edwards Jr TC, Graham CH, Pearman PB, Svenning J-C. New trends in species distribution modelling. Ecography. 2010;33(6):985-9. doi: 10.1111/j.1600-0587.2010.06953.x.

2. Domisch S, Friedrichs M, Hein T, Borgwardt F, Wetzig A, Jähnig SC, et al. Spatially explicit species distribution models: A missed opportunity in conservation planning? Divers Distrib. 2019;25(5):758-69. doi: 10.1111/ddi.12891.

3. Phillips SJ, Anderson RP, Schapire RE. Maximum entropy modeling of species geographic distributions. Ecological Modelling. 2006;190(3–4):231-59. doi: 10.1016/j.ecolmodel.2005.03.026.

4. Anderson RP. Harnessing the world's biodiversity data: promise and peril in ecological niche modeling of species distributions. Annals of the New York Academy of Sciences. 2012;1260(1):66-80. doi: 10.1111/j.1749-6632.2011.06440.x.

5. Guisan A, Tingley R, Baumgartner JB, Naujokaitis-Lewis I, Sutcliffe PR, Tulloch AIT, et al. Predicting species distributions for conservation decisions. Ecology Letters. 2013;16(12):1424-35. doi: 10.1111/ele.12189.

6. Fourcade Y, Besnard AG, Secondi J. Paintings predict the distribution of species, or the challenge of selecting environmental predictors and evaluation statistics. Global Ecology and Biogeography. 2018;27(2):245-56. doi: 10.1111/geb.12684.

7. Jiménez- Valverde A, Lobo JM, Hortal J. Not as good as they seem: the importance of concepts in species distribution modelling. Divers Distrib. 2008;14(6):885-90. doi: 10.1111/j.1472-4642.2008.00496.x.

8. Benito BM, Cayuela L, Albuquerque FS. The impact of modelling choices in the predictive performance of richness maps derived from species-distribution models: guidelines to build better diversity models. Methods Ecol Evol. 2013;4(4):327-35. doi: 10.1111/2041-210x.12022.

9. Visconti P, Di Marco M, Álvarez- Romero J, Januchowski- Hartley S, Pressey R, Weeks R, et al. Effects of errors and gaps in spatial data sets on assessment of conservation progress. Conservation Biology. 2013;27(5):1000-10. doi: 10.1111/cobi.12095.

10. Greaves GJ, Mathieu R, Seddon PJ. Predictive modelling and ground validation of the spatial distribution of the New Zealand long-tailed bat (Chalinolobus tuberculatus). Biol Conserv. 2006;132(2):211-21. doi: 10.1016/j.biocon.2006.04.016.

11. Hipólito J, Hasui É, Viana BF. Solving problems involving the distribution of a species of unknown distribution via ecological niche modeling. Nat Conservacao. 2015;13(1):15-23. doi: 10.1016/j.ncon.2015.03.001.

12. Carraro L, Mächler E, Wüthrich R, Altermatt F. Environmental DNA allows upscaling spatial patterns of biodiversity in freshwater ecosystems. Nature Communications. 2020;11(1):3585. doi: 10.1038/s41467-020-17337-8.

13. Cubaynes HC, Fretwell PT, Bamford C, Gerrish L, Jackson JA. Whales from space: Four mysticete species described using new VHR satellite imagery. Marine Mammal Science. 2019;35(2):466-91. doi: 10.1111/mms.12544.

14. Laiolo P. The emerging significance of bioacoustics in animal species conservation. Biol Conserv. 2010;143(7):1635-45. doi: http://doi.org/10.1016/j.biocon.2010.03.025.

15. Schnitzler H-U, Moss CF, Denzinger A. From spatial orientation to food acquisition in echolocating bats. Trends Ecol Evol. 2003;18(8):386-94. doi: 10.1016/s0169-5347(03)00185-x.

16. Parsons S, Szewczak J. Detecting, recording and analysing the vocalisations of bats. Ecological and Behavioral Methods for the Study of Bats [2nd Ed]: Johns Hopkins University Press; 2009. p. 91-111.

17. Barataud M, Giosa S, Leblanc F, Rufray V, Disca T, Tillon L, et al. Identification et écologie acoustique des chiroptères de Guyane Française. Le Rhinolophe. 2013;19:103-45.

18. Arias-Aguilar A, Hintze F, Aguiar LMS, Rufray V, Bernard E, Pereira MJR. Who's calling? Acoustic identification of Brazilian bats. Mammal Research. 2018;63(3):231-53. doi: 10.1007/s13364-018-0367-z.

19. Hintze F, Arias-Aguilar A, Dias-Silva L, Delgado-Jaramillo M, Silva CR, Jucá T, et al. Molossid unlimited: extraordinary extension of range and unusual vocalization patterns of the bat, Promops centralis. J Mammal. 2020;101(2):417-32. doi: 10.1093/jmammal/gyz167.

20. Barquez RMD, Ojeda MM, Ricardo A. Mamíferos de Argentina: sistemática y distribución: SAREM; 2006.

21. Gardner AL. Mammals of South America, volume 1: marsupials, xenarthrans, shrews, and bats: University of Chicago Press; 2008.

22. Peterson AT, Soberón J, Pearson RG, Anderson RP, Martínez-Meyer E, Nakamura M, et al. Ecological niches and geographic distributions (MPB-49): Princeton University Press; 2011.

23. Brown JL. SDMtoolbox: a python- based GIS toolkit for landscape genetic, biogeographic and species distribution model analyses. Methods Ecol Evol. 2014;5(7):694-700. doi: doi:10.1111/2041-210X.12200.

24. Fick SE, Hijmans RJ. WorldClim 2: new 1-km spatial resolution climate surfaces for global land areas. International Journal of Climatology. 2017;37(12):4302-15. doi: 10.1002/joc.5086.

25. Coxen CL, Frey JK, Carleton SA, Collins DP. Species distribution models for a migratory bird based on citizen science and satellite tracking data. Global Ecology and Conservation. 2017;11:298-311. doi: https://doi.org/10.1016/j.gecco.2017.08.001.

26. Hidalgo-Mihart MG, Cantú-Salazar L, González-Romero A, López-González CA. Historical and present distribution of coyote (Canis latrans) in Mexico and Central America. Journal of Biogeography. 2004;31(12):2025-38. doi: doi:10.1111/j.1365-2699.2004.01163.x.

27. Pearson RG, Raxworthy CJ, Nakamura M, Townsend PA. Predicting species distributions from small numbers of occurrence records: a test case using cryptic geckos in Madagascar. Journal of Biogeography. 2007;34(1):102-17. doi: doi:10.1111/j.1365-2699.2006.01594.x.

28. Radosavljevic A, Anderson RP. Making better Maxent models of species distributions: complexity, overfitting and evaluation. Journal of Biogeography. 2014;41(4):629-43. doi: 10.1111/jbi.12227.

29. Fourcade Y, Engler JO, Rödder D, Secondi J. Mapping Species Distributions with MAXENT Using a Geographically Biased Sample of Presence Data: A Performance Assessment of Methods for Correcting Sampling Bias. PLoS ONE. 2014;9(5):e97122. doi: 10.1371/journal.pone.0097122.

30. Arino O, Ramos Perez JJ, Kalogirou V, Bontemps S, Defourny P, Van Bogaert E. Global land cover map for 2009 (GlobCover 2009). ESA & UCL. 2012.

31. Snedecor G, Cochran W. Statistical methods. VII Edition ed. Iowa: Iowa State University Press; 1980.

32. Phillips SJ, Dudík M, Elith J, Graham CH, Lehmann A, Leathwick J, et al. Sample selection bias and presence-only distribution models: implications for background and pseudo-absence data. Ecological Applications. 2009;19(1):181-97. doi: 10.1890/07-2153.1.

33. Merow C, Smith MJ, Silander JA. A practical guide to MaxEnt for modeling species' distributions: what it does, and why inputs and settings matter. Ecography. 2013;36(10):1058-69. doi: doi:10.1111/j.1600-0587.2013.07872.x.

34. Liu C, Newell G, White M. On the selection of thresholds for predicting species occurrence with presence-only data. Ecology and Evolution. 2016;6(1):337-48. doi: doi:10.1002/ece3.1878.

35. Liu C, White M, Newell G. Selecting thresholds for the prediction of species occurrence with presence-only data. Journal of Biogeography. 2013;40(4):778-89. doi: 10.1111/jbi.12058.

36. Jiménez-Valverde A, Lobo JM. Threshold criteria for conversion of probability of species presence to either–or presence–absence. Acta Oecologica. 2007;31(3):361-9. doi: https://doi.org/10.1016/j.actao.2007.02.001.

37. Brooner W. Land-use map accuracy criteria. Photogrammetric Engineering and Remote Sensing. 1976;42(5):671-7.

38. Allouche O, Tsoar A, Kadmon R. Assessing the accuracy of species distribution models: prevalence, kappa and the true skill statistic (TSS). Journal of Applied Ecology. 2006;43(6):1223-32. doi: 10.1111/j.1365-2664.2006.01214.x.

39. Cohen J. A Coefficient of Agreement for Nominal Scales. Educational and Psychological Measurement. 1960;20(1):37-46. doi: 10.1177/001316446002000104.

40. Mingyang L, Yunwei J, Kumar S, Stohlgren TJ. Modeling potential habitats for alien species Dreissena polymorpha in Continental USA. Acta Ecologica Sinica. 2008;28(9):4253-8. doi: https://doi.org/10.1016/S1872-2032(08)60080-3.

41. Peirce CS. The numerical measure of the success of predictions. Science. 1884;ns-4(93):453-4. doi: 10.1126/science.ns-4.93.453-a.

42. Wunderlich RF, Lin Y-P, Anthony J, Petway JR. Two alternative evaluation metrics to replace the true skill statistic in the assessment of species distribution models. Nature Conservation. 2019;35. doi: 10.3897/natureconservation.35.33918.

43. Adams AM, Jantzen MK, Hamilton RM, Fenton MB. Do you hear what I hear? Implications of detector selection for acoustic monitoring of bats. Methods Ecol Evol. 2012;3(6):992-8. doi: http://dx.doi.org/10.1111/j.2041-210X.2012.00244.x.

44. Ratcliffe JM, Jakobsen L. Don't believe the mike: behavioural, directional, and environmental impacts on recorded bat echolocation call measures. Canadian Journal of Zoology. 2018:283-8. doi: 10.1139/cjz-2017-0219.

45. Lloyd A, Law B, Goldingay R. Bat activity on riparian zones and upper slopes in Australian timber production forests and the effectiveness of riparian buffers. Biol Conserv. 2006;129(2):207-20. doi: 10.1016/j.biocon.2005.10.035.

46. Jung K, Molinari J, Kalko EKV. Driving Factors for the Evolution of Species-Specific Echolocation Call Design in New World Free-Tailed Bats (Molossidae). PLoS ONE. 2014;9(1):e85279. doi: 10.1371/journal.pone.0085279.

47. Jung K, Kalko EK, Helversen Ov. Echolocation calls in Central American emballonurid bats: signal design and call frequency alternation. J Zool. 2007;272(2):125-37. doi: 10.1111/j.1469-7998.2006.00250.x.

48. López-Baucells A, Rocha R, Bobrowiec P, Bernard E, Palmeirim J, Meyer C. Field Guide to Amazonian Bats. López-Baucells A, Rocha R, Bobrowiec P, Bernard E, Palmeirim J, Meyer C, editors. Manaus, Brazil: Editora INPA; 2016. 168 p.

49. Sikes RS, II JAB, Byman D, Danielson BJ, Eggleston J, Gannon MR, et al. Guidelines of the American Society of Mammalogists for the use of wild mammals in research and education. J Mammal. 2016;97(3):663-88. Epub 2016/05/28. doi: 10.1093/jmammal/gyw078. PubMed PMID: 29692469.

50. Branco P, Torgo L, Ribeiro RP. A Survey of Predictive Modeling on Imbalanced Domains. ACM Comput Surv. 2016;49(2):Article 31. doi: 10.1145/2907070.

51. Daskalaki S, Kopanas I, Avouris N. EVALUATION OF CLASSIFIERS FOR AN UNEVEN CLASS DISTRIBUTION PROBLEM. Applied Artificial Intelligence. 2006;20(5):381-417. doi: 10.1080/08839510500313653.

52. Bernard E, Aguiar LMS, Machado RB. Discovering the Brazilian bat fauna: a task for two centuries? Mamm Rev. 2011;41(1):23-39. doi: 10.1111/j.1365-2907.2010.00164.x.

53. Delgado-Jaramillo M, Aguiar LMS, Machado RB, Bernard E. Assessing the distribution of a species-rich group in a continental-sized megadiverse country: Bats in Brazil. Divers Distrib. 2020;n/a(n/a). doi: 10.1111/ddi.13043.

54. Hertzog LR, Besnard A, Jay-Robert P. Field validation shows bias-corrected pseudo-absence selection is the best method for predictive species-distribution modelling. Divers Distrib. 2014;20(12):1403-13. doi: 10.1111/ddi.12249.

55. Giné GAF, Faria D. Combining species distribution modeling and field surveys to reappraise the geographic distribution and conservation status of the threatened thin-spined porcupine (Chaetomys subspinosus). PLoS ONE. 2018;13(11):e0207914. doi: 10.1371/journal.pone.0207914.

56. Rebelo H, Jones G. Ground validation of presence-only modelling with rare species: a case study on barbastelles Barbastella barbastellus (Chiroptera: Vespertilionidae). Journal of Applied Ecology. 2010;47(2):410-20. doi: 10.1111/j.1365-2664.2009.01765.x.

57. Razgour O, Rebelo H, Di Febbraro M, Russo D. Painting maps with bats: species distribution modelling in bat research and conservation. Hystrix, the Italian Journal of Mammalogy. 2016;27(1). doi: 10.4404/hystrix-27.1-11753.

58. Kramer-Schadt S, Niedballa J, Pilgrim JD, Schröder B, Lindenborn J, Reinfelder V, et al. The importance of correcting for sampling bias in MaxEnt species distribution models. Divers Distrib. 2013;19(11):1366-79. doi: https://doi.org/10.1111/ddi.12096.

59. Ferri C, Hernández-Orallo J, Modroiu R. An experimental comparison of performance measures for classification. Pattern Recognition Letters. 2009;30(1):27-38. doi: https://doi.org/10.1016/j.patrec.2008.08.010.

60. West AM, Kumar S, Brown CS, Stohlgren TJ, Bromberg J. Field validation of an invasive species Maxent model. Ecological Informatics. 2016;36:126-34. doi: https://doi.org/10.1016/j.ecoinf.2016.11.001.

61. Hood CS, Jones JK. Noctilio leporinus. Mamm Species. 1984;(216):1-7.

62. Yancey FD, II, Goetze JR, Jones C. Saccopteryx leptura. Mamm Species. 1998;(582):1-3. doi: 10.2307/3504379.

63. Wilson JW, Sexton JO, Todd Jobe R, Haddad NM. The relative contribution of terrain, land cover, and vegetation structure indices to species distribution models. Biol Conserv. 2013;164:170-6. doi: https://doi.org/10.1016/j.biocon.2013.04.021.

64. Beck J, Böller M, Erhardt A, Schwanghart W. Spatial bias in the GBIF database and its effect on modeling species' geographic distributions. Ecological Informatics. 2014;19:10-5. doi: https://doi.org/10.1016/j.ecoinf.2013.11.002.

Supporting information captions

S1 Table. Location of the 1277 single records of the *Noctilio leporinus, Promops centralis, Promops nasutus, Pteronotus gymnonotus, Pteronotus personatus* and *Saccopteryx leptura,* gathered after our bibliographic revision.

S2 Table. Prediction variables used in this study for the distribution modelling of six neotropical bat species in northeastern Brazil.

S3 Table. Presence/absence obtained in the acoustic monitoring of 129 acoustic sampling points used for the validation of SDM's of six neotropical bat species in northeastern Brazil.

S4 Table. Theoretical model evaluation scores, validation confusion matrix, and performance scores of the 144 binary distribution models of six neotropical bat species validated with field acoustics sampling in northeastern Brazil.

S5 Maps. Field validation results for the binary maps with the highest accuracy, precision, sensitivity, specificity, gmean, and f-score scores for the six studied species in northeastern Brazil.

S6 Table. Average ± standard deviation scores of accuracy, precision, sensitivity, specificity, g-mean, and f-score of the three thresholds tested for SDMs of six neotropical bat species after a field validation in northeastern Brazil using bioacoustics. Kruskal-Wallis test results for the comparisons between the thresholds' performance scores is also presented. The Mann-Whitney pairwise post hoc test results are presented by letters next to average ± standard deviation (different letters indicate significant differences between groups, $p < 0.05$).

Figure 1

Click here to access/download;Figure;Fig1.tif

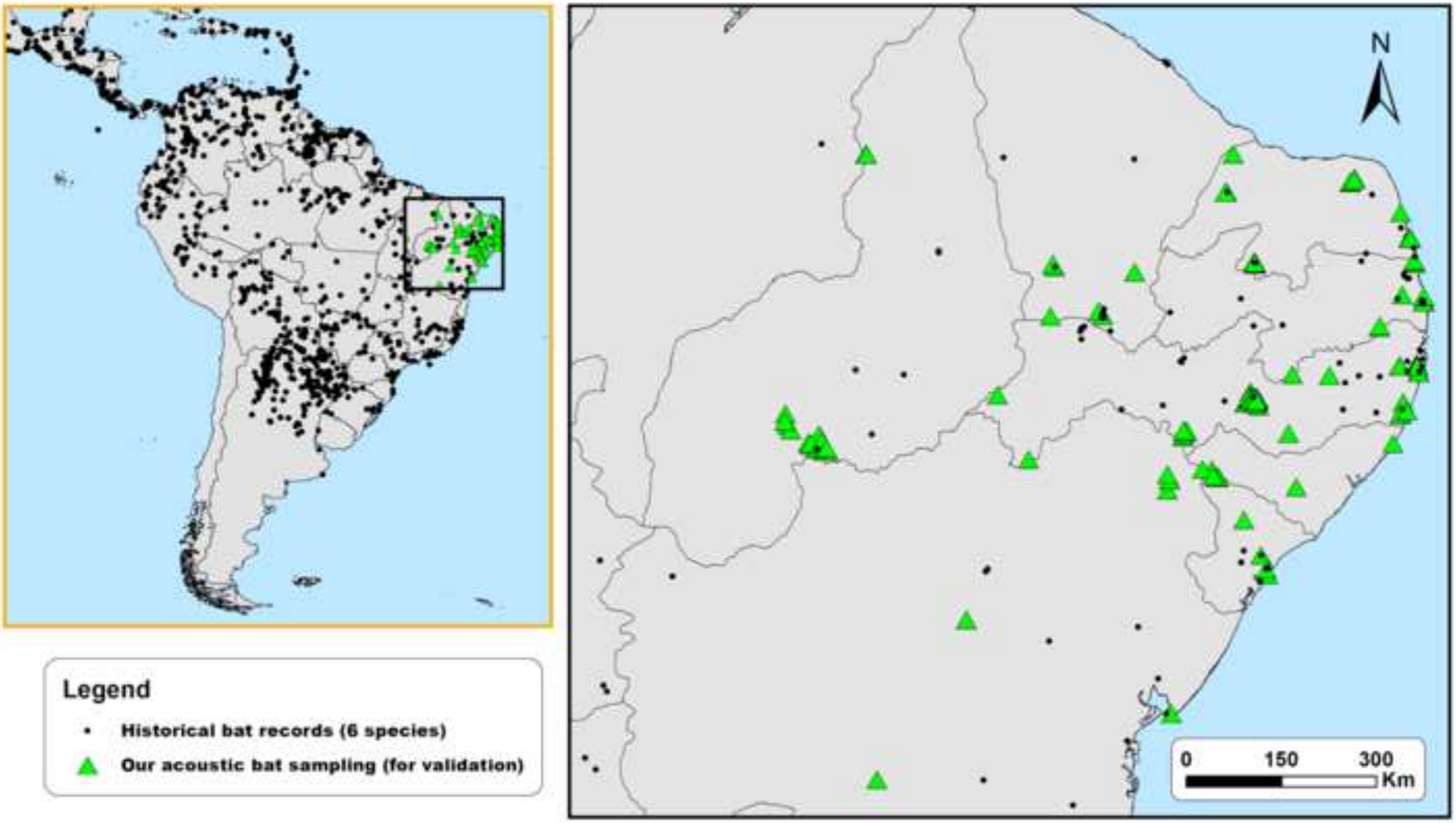

Background

Figure 2

Click here to access/download;Figure;Fig2.tiff

Allsp_thresholds.tiff

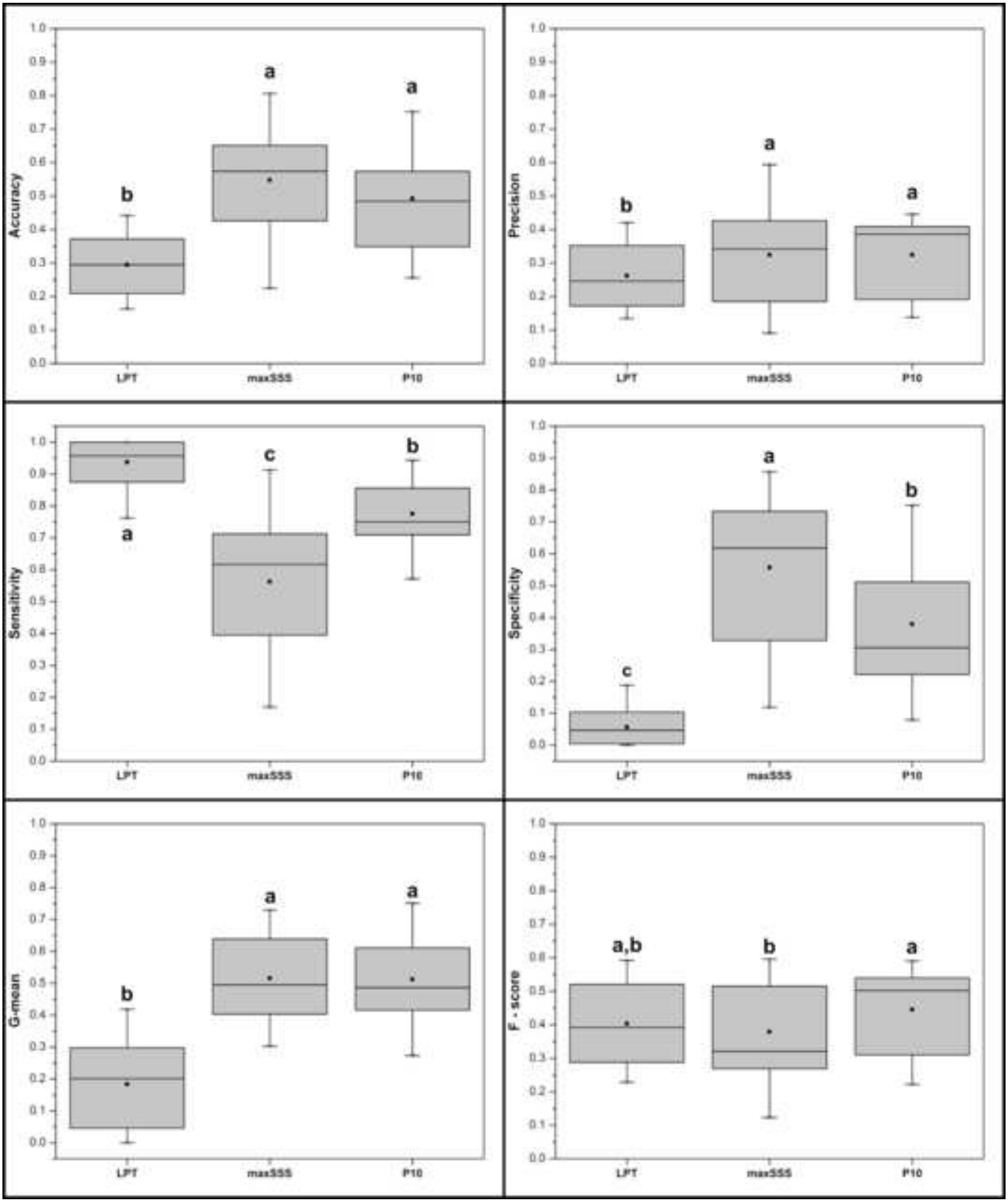

Figure 3

Click here to access/download;Figure;Fig3.tiff

Nlep_thresholds.tiff

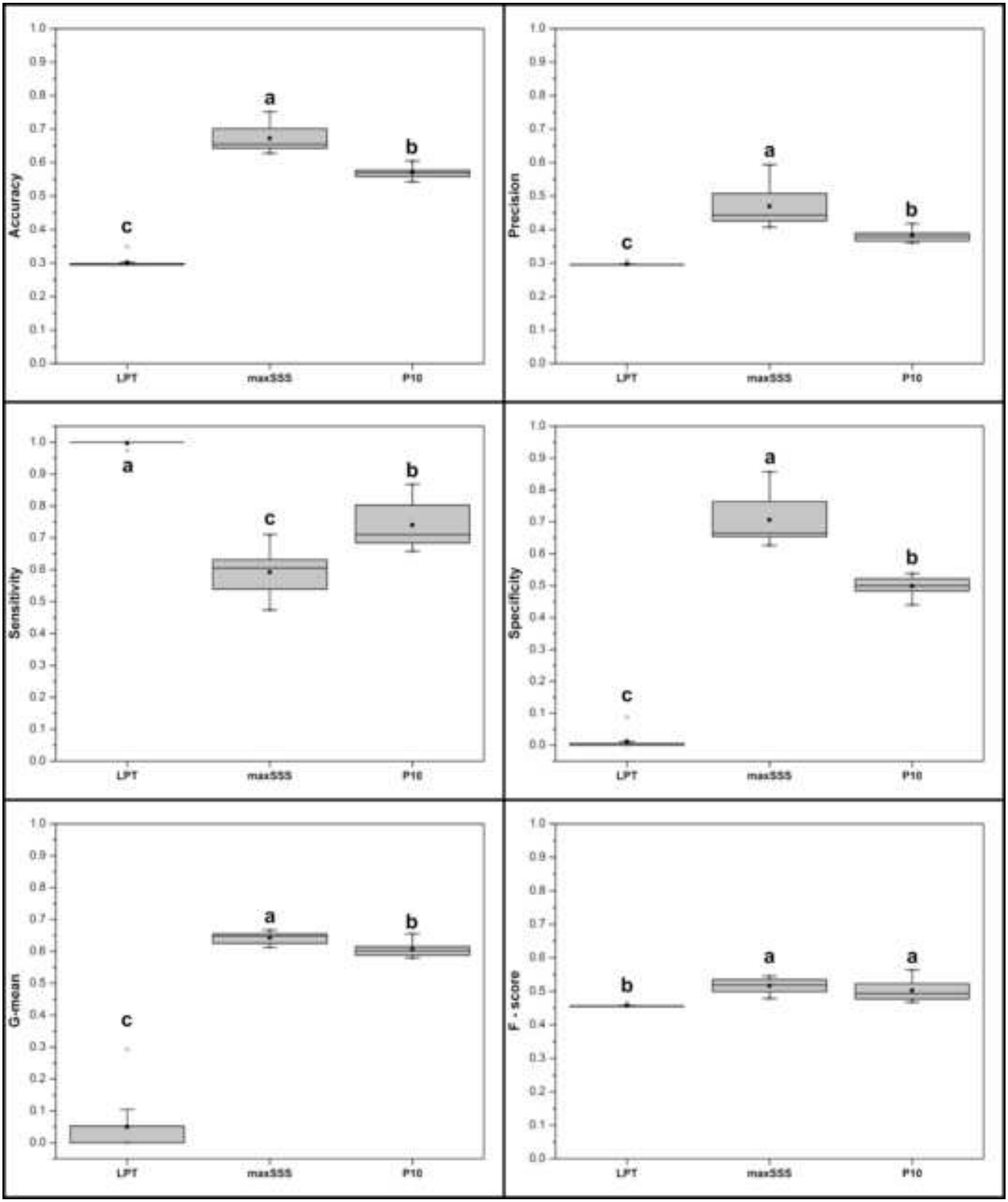

Figure 4

Click here to access/download;Figure;Fig4.tiff

Pcen_Thresholds.tiff

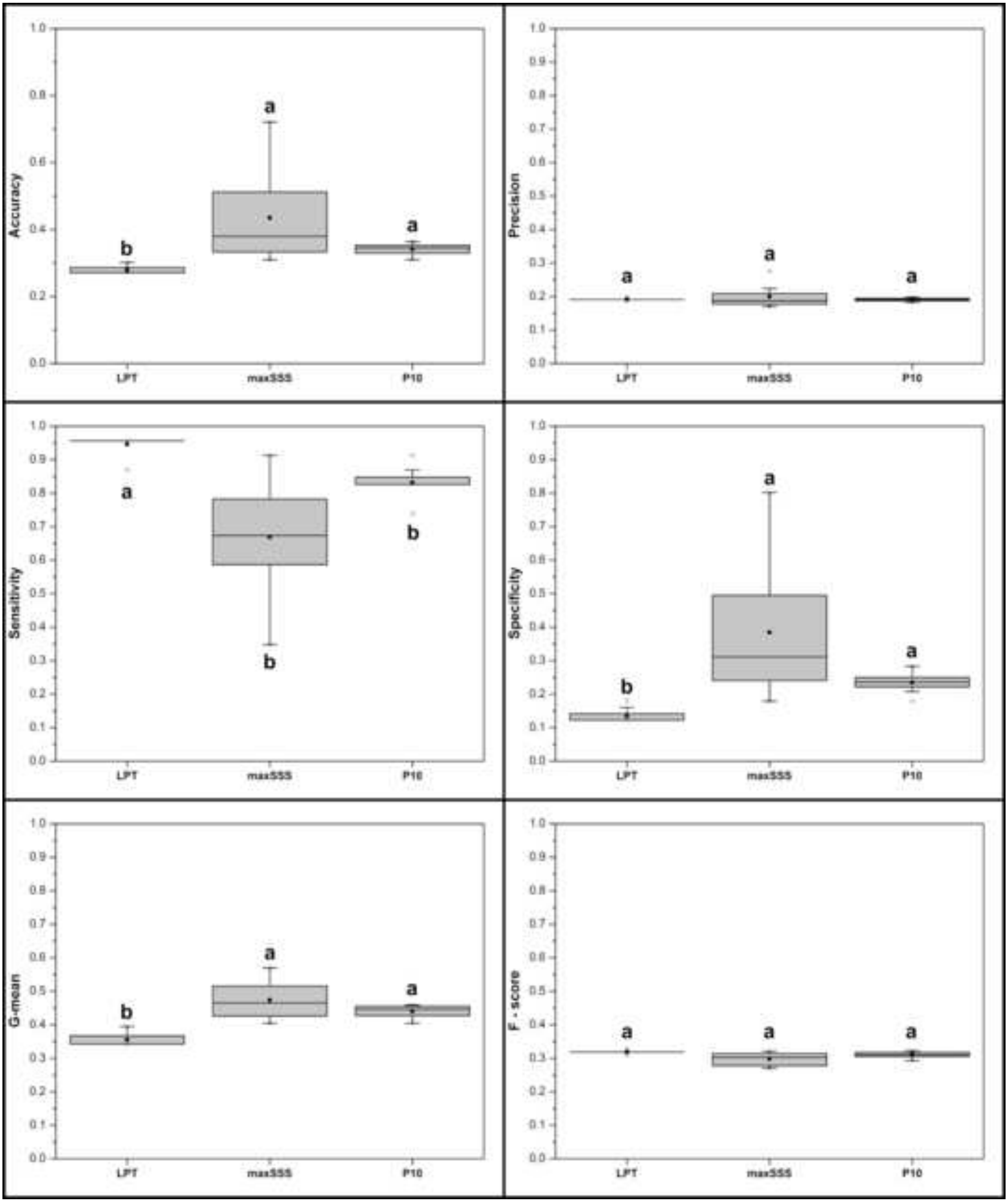

Figure 5

Click here to access/download;Figure;Fig5.tiff

Pgym_thresholds.tiff

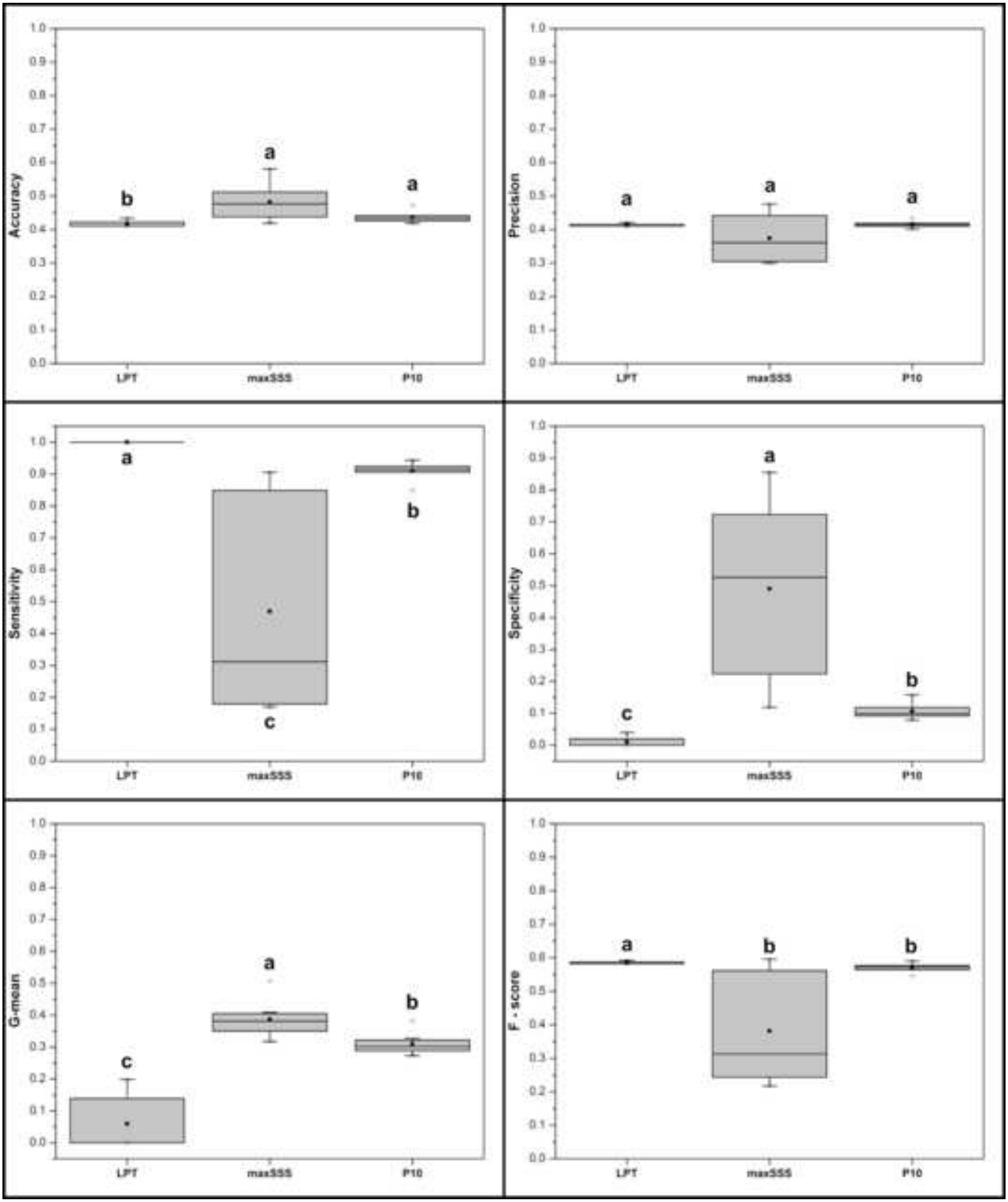

 Click here to access/download;Figure;Fig6.tiff

Pnas_Threshold.tiff

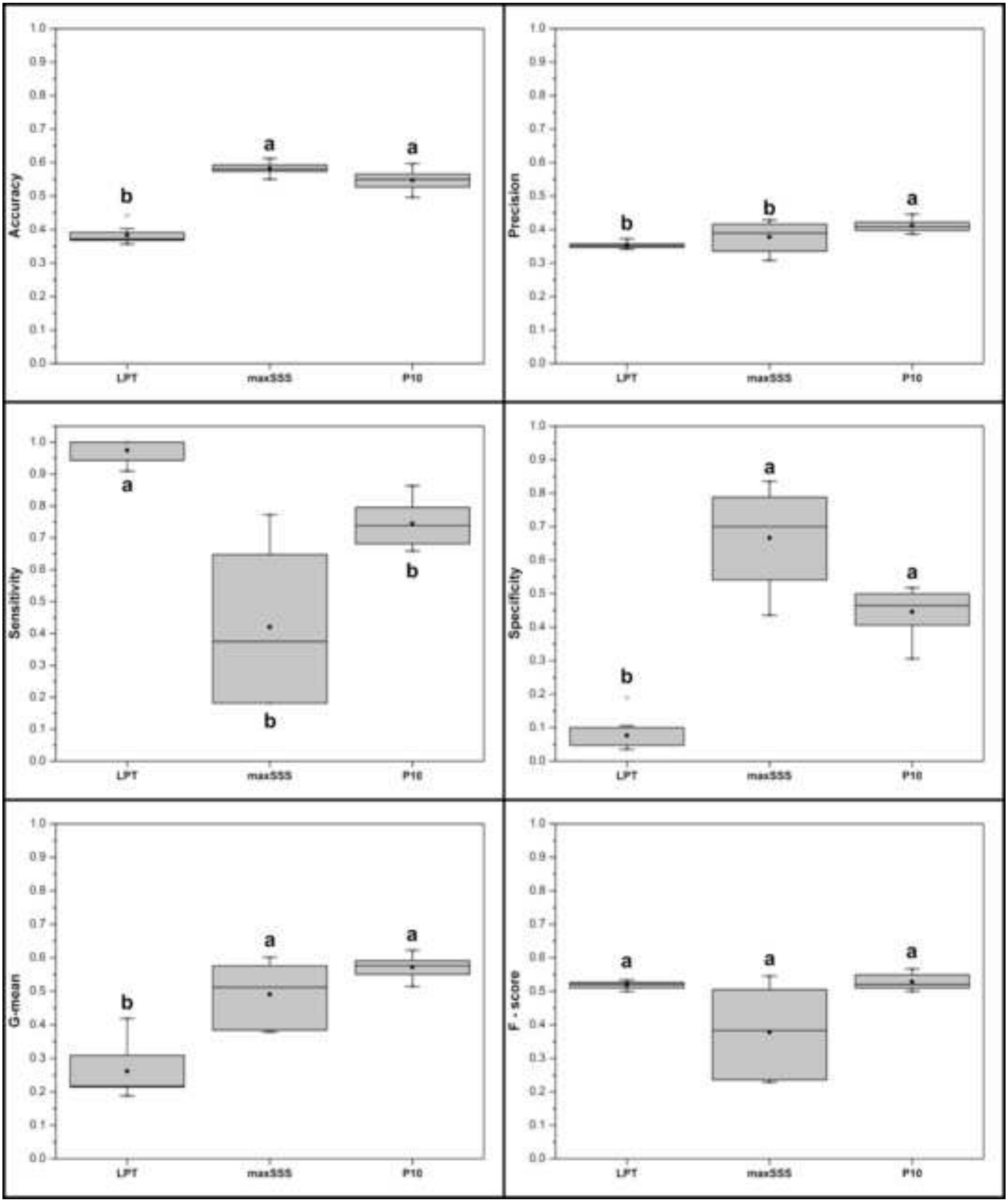

Pper_Thresholds.tiff

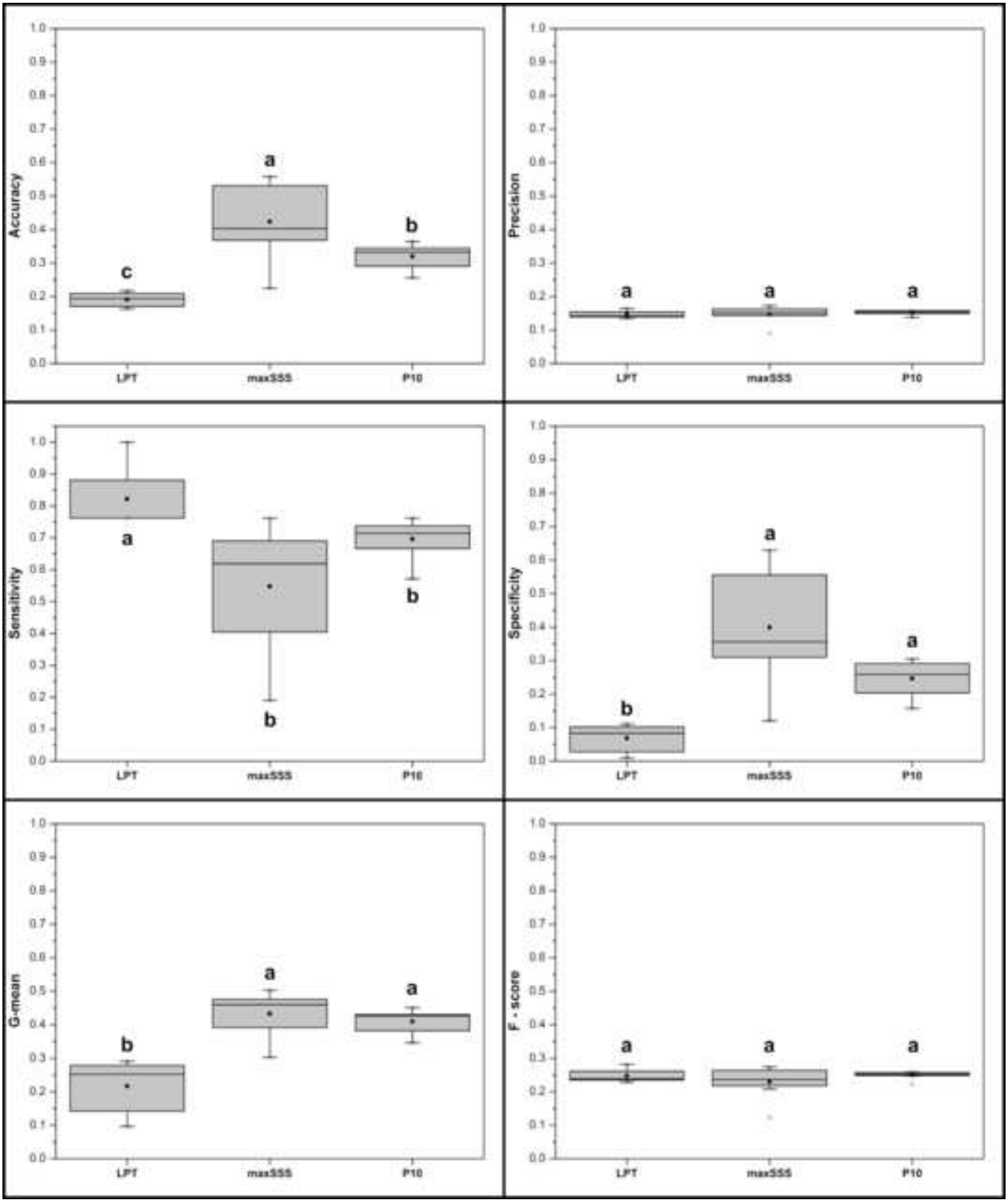

Click here to access/download;Figure;Fig8.tiff

Slep_Thresholds.tiff

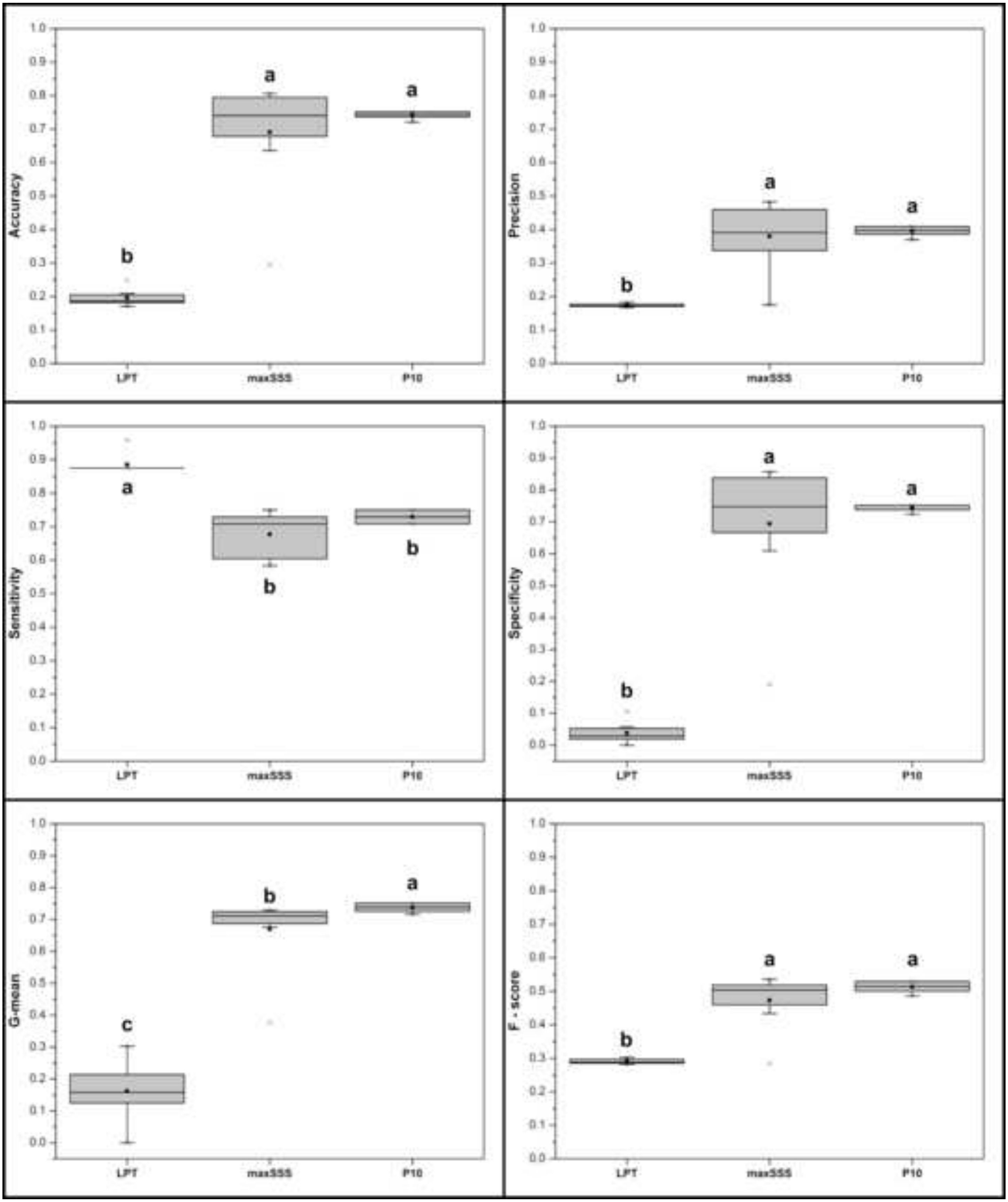

Click here to access/download;Figure;Fig9.tif

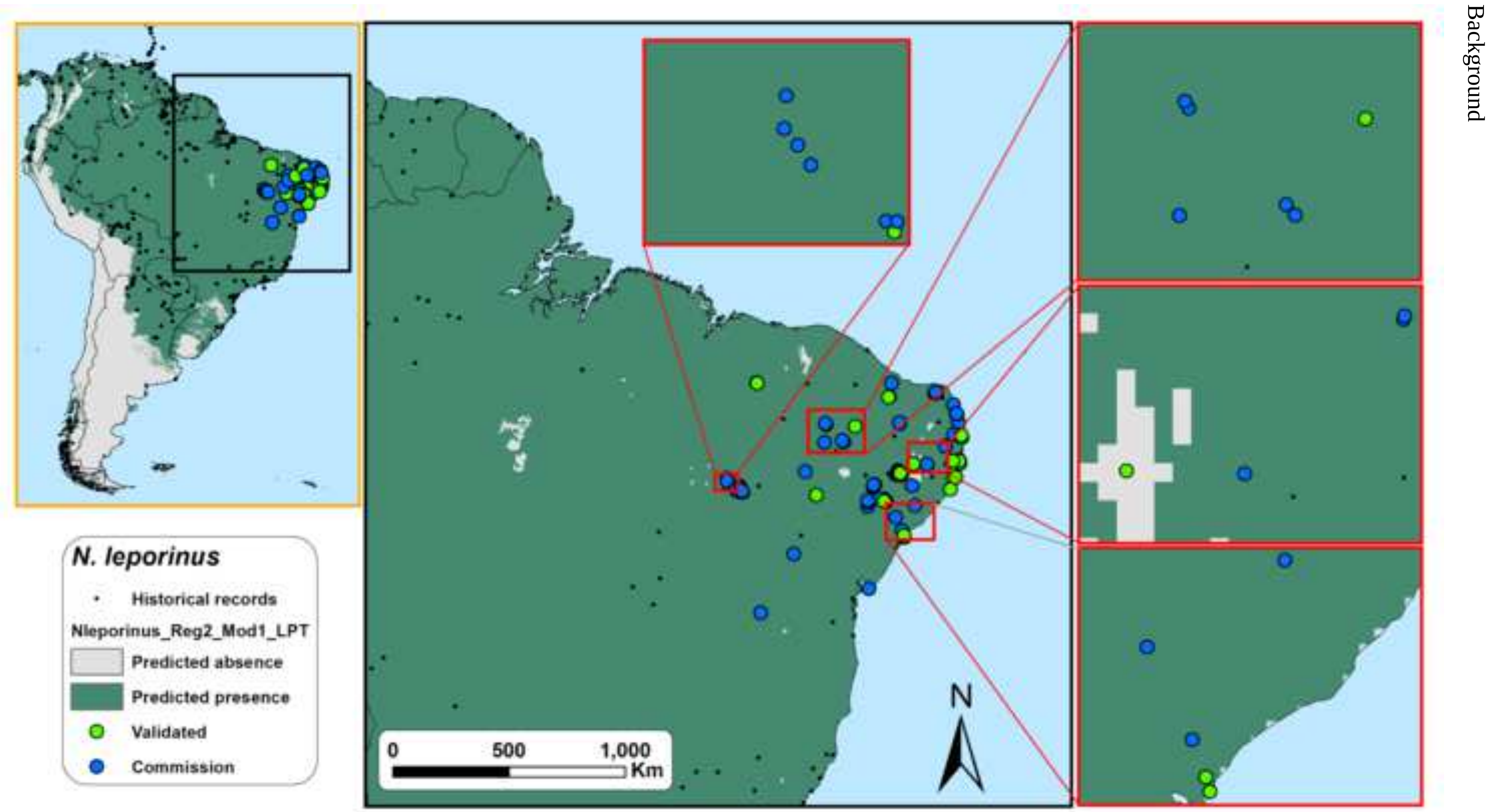

Click here to access/download;Figure;Fig10.tif

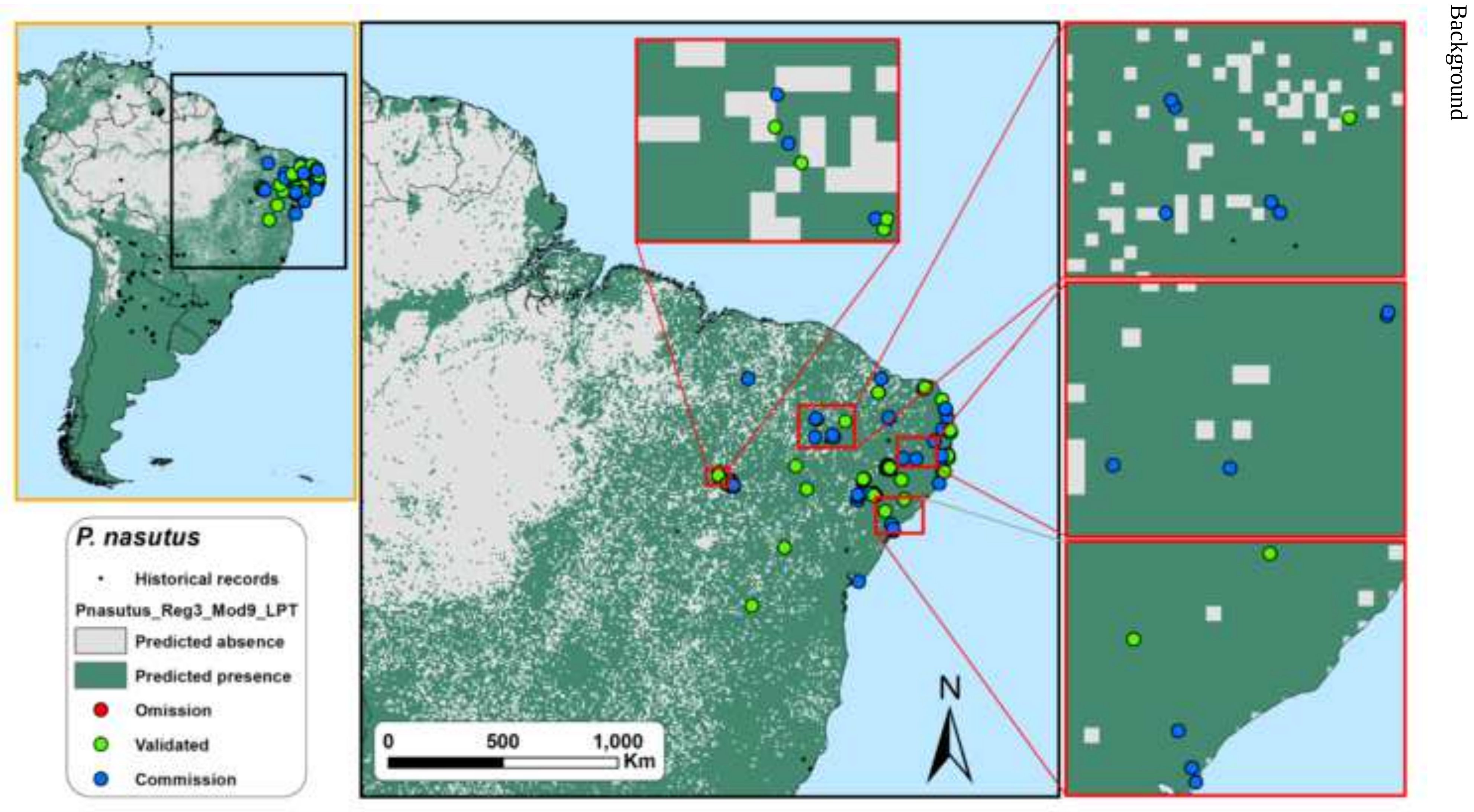

Figure 11

Click here to access/download;Figure;Fig11.tif

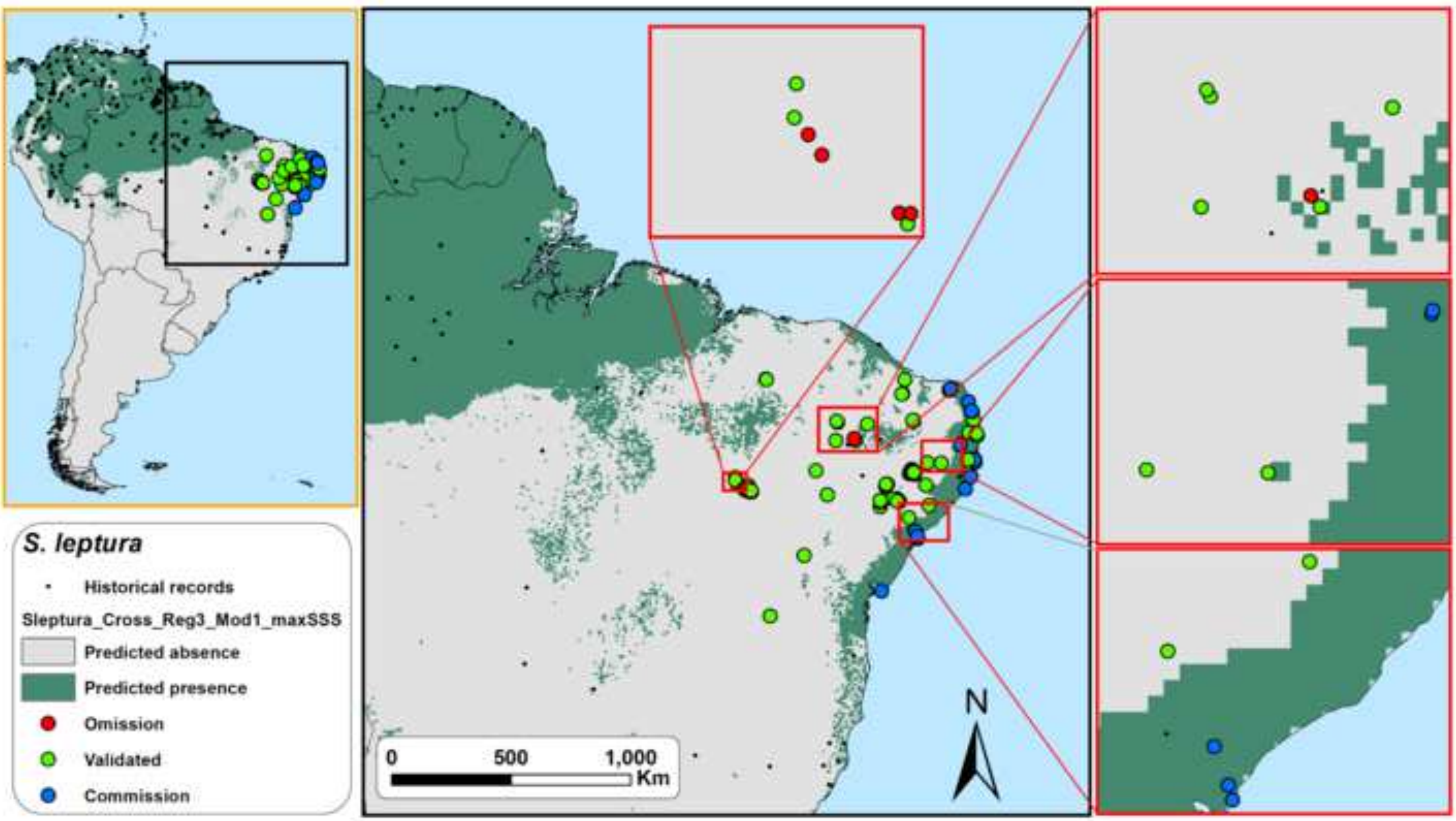

Background

S1 Table

Click here to access/download
Supporting Information
S1_table.xlsx

S2 Table

Click here to access/download
Supporting Information
S2_Table.xlsx

S3 Table

Click here to access/download
Supporting Information
S3_Table.xlsx

S4 Table

Click here to access/download
Supporting Information
S4_Table.xlsx

S5 Maps

Click here to access/download
Supporting Information
S5_Maps.pdf

S6 Table

Click here to access/download
Supporting Information
S6_Table.docx
