# Supplementary material for: Bioacoustics for in situ validation of species distribution modelling: An example with bats in Brazil
Source: PLoS One. 2021 Oct 20;16(10):e0248797. doi: 10.1371/journal.pone.0248797 (PMC8528307; doi:10.1371/journal.pone.0248797)
Supplement: Attachment — Submitted filename: Response to Reviewers.pdf [file pone.0248797.s008.pdf]

Centro de Biociências **Universidade Federal de Pernambuco**

From: Frederico Hintze

To: Dr. Daniel de Paiva Silva – Academic Editor, PLOS One

Ref.: PONE-D-21-07379

Recife, August 13th 2021.

Dear Dr. Daniel de Paiva Silva,

I would like to resubmit for possible publication in PLOS One our manuscript "Bioacoustics for in situ validation of species distribution modelling: An example with bats in Brazil", PONE-D-21-07379, whose authors are Ricardo B. Machado, Enrico Bernard, and myself as first author.

Thank you very much for the review of the manuscript, the comments and suggestions you made. We carefully reviewed and addressed all notes and change proposals made by you and the reviewers. Below you will find a detailed list of comments and corrections made based on the points you and reviewers raised. Please let us know if any other information is necessary.

Best regards,

Frederico Hintze
Laboratório de Ciência Aplicada à Conservação da Biodiversidade
Departamento de Zoologia, Universidade Federal de Pernambuco
Rua Professor Nelson Chaves s/n, Cidade Universitária, Recife, PE 50670-420 Brazil
Phone/fax (+55) 81 2126 8353 e-mail: fredhintze@gmail.com

Academic Editor's comments

1. Please ensure that your manuscript meets PLOS ONE's style requirements, including those for file naming. The PLOS ONE style templates can be found at

https://journals.plos.org/plosone/s/file?id=wjVg/PLOSOne_formatting_sample_main_body.pdf

and

https://journals.plos.org/plosone/s/file?id=ba62/PLOSOne_formatting_sample_title_authors_affiliations.pdf

Answer: We formatted the new manuscript accordingly to the style templates.

2. Please ensure that you refer to Figure 9 in your text as, if accepted, production will need this reference to link the reader to the figure.

Answer: We corrected it. The Figure 9 is now Figure 12.

3. We note that Figures 1,9,10,11 and A-U in your submission contain map images which may be copyrighted. All PLOS content I s published under the Creative Commons Attribution License (CC BY 4.0), which means that the manuscript, images, and Supporting Information files will be freely available online, and any third party is permitted to access, download, copy, distribute, and use these materials in any way, even commercially, with proper attribution. For these reasons, we cannot publish previously copyrighted maps or satellite images created using proprietary data, such as Google software (Google Maps, Street View, and Earth). For more information, see our copyright guidelines: http://journals.plos.org/plosone/s/licenses-and-copyright.

We require you to either (1) present written permission from the copyright holder to publish these figures specifically under the CC BY 4.0 license, or (2) remove the figures from your submission:

1. **You may seek permission from the original copyright holder of Figures 1,9,10,11 and A-U to publish the content specifically under the CC BY 4.0 license.**

   We recommend that you contact the original copyright holder with the Content Permission Form (http://journals.plos.org/plosone/s/file?id=7c09/content-permission-form.pdf) and the following text:

   "I request permission for the open-access journal PLOS ONE to publish XXX under the Creative Commons Attribution License (CCAL) CC BY 4.0 (http://creativecommons.org/licenses/by/4.0/). Please be aware that this license allows unrestricted use and distribution, even commercially, by third parties. Please reply and provide explicit written permission to publish XXX under a CC BY license and complete the attached form."

   Please upload the completed Content Permission Form or other proof of granted permissions as an "Other" file with your submission.

In the figure caption of the copyrighted figure, please include the following text: "Reprinted from [ref] under a CC BY license, with permission from [name of publisher], original copyright [original copyright year]."

2. **If you are unable to obtain permission from the original copyright holder to publish these figures under the CC BY 4.0 license or if the copyright holder's requirements are incompatible with the CC BY 4.0 license, please either i) remove the figure or ii) supply a replacement figure that complies with the CC BY 4.0 license. Please check copyright information on all replacement figures and update the figure caption with source information. If applicable, please specify in the figure caption text when a figure is similar but not identical to the original image and is therefore for illustrative purposes only.**

The following resources for replacing copyrighted map figures may be helpful:

USGS National Map Viewer (public domain): http://viewer.nationalmap.gov/viewer/

The Gateway to Astronaut Photography of Earth (public domain): http://eol.jsc.nasa.gov/sseop/clickmap/

Maps at the CIA (public domain): https://www.cia.gov/library/publications/the-world-factbook/index.html and https://www.cia.gov/library/publications/cia-maps-publications/index.html

NASA Earth Observatory (public domain): http://earthobservatory.nasa.gov/

Landsat: http://landsat.visibleearth.nasa.gov/

USGS EROS (Earth Resources Observatory and Science (EROS) Center) (public domain): http://eros.usgs.gov/#

Natural Earth (public domain): http://www.naturalearthdata.com/

Answer: We already created our maps using Natural Earth's imagery of South America, so there are no copyright infringements on the figures. Nevertheless, now we cited Natural Earth in each caption: "Made with Natural Earth. Free vector and raster map data @ naturalearthdata.com.".

Additional Editor Comments

Dear Hintze et al.,

After two independent reviews, I believe your manuscript may be accepted for publication after a major review when you will have the possibility to take care of the issues raised by both reviewers. The reviewers decided on a major and a minor review. Although both reviewers raised important issues, please take special care regarding the issues raised by reviewer #2, who raised important issues related to your writing and requested you to be more explicit in the relation between the study's goals and results.

Considering the pandemic situation in Brazil, I believe a three-month (August 1st 2021) period will be more than enough for you to deliver the revised version of your text. By that time, along with the revised version of your text, please do not forget to prepare a rebuttal letter where you will explain all the decisions you took regarding the issues raised by the reviewers. Do not hesitate to resubmit earlier in case you are able to. Nonetheless, in case you need more time, please let me know.

Sincerely,

Daniel Silva

Answer: We carefully reviewed and addressed all notes and change proposals made by you and the reviewers. Below you will find a detailed list of comments and corrections made based on the points reviewers raised.

Reviewer 1’ comments

In this manuscript, the authors use a sound presence-only database of neotropical bats to build presence-absence maps based on species distribution modeling (SDM) techniques, specifically using MaXent. The authors test distinct combinations of parameters and validate the dataset using an impressive independent dataset, obtained with acoustic monitoring. I appreciate the introduction and believe that sampling procedures and methodological procedures are adequate for the study. The results section seems to have some results missing, and the figures could have some more editing to facilitate reading the many findings of this study. Finally, I believe that the discussion is fine in content, but could be organized to improve interpretations, with a clear take-home message for the broad public. One last point is that although the title contains bioacoustics, I missed seeing some discussion on the cost-benefit of acoustic and non-acoustic sampling to validate SDM. This diligent study will be a good contribution to the field. Bellow, I provide some suggestions and commentaries through the manuscript to be considered before publication.

Abstract

1. **“L. 15: estimates of species diversity?”**

   Answer: Species distribution estimates. We changed the text from “Such modelling can be beneficial for large-scale approaches and estimates (…)” to “Such modelling can be beneficial for large-scale approaches and species distribution estimates (…)”.

Introduction

2. **L. 41-46: “some sentences are repeated from the abstract. Consider rephrasing them.”**

   Answer: We rephrased the sentence to “This is an advantage for large-scale approaches and species distribution estimates, since few parts of our planet have been adequately sampled”.

3. **L. 55: “Is there a spatial modeling science? Maybe spatial modeling, or just modeling, would be the method to make science (ecology) more applied, where errors should be minimized.”**

   Answer: We changed the text to “spatial modelling”.

4. **L. 74: “I think the goal is to evaluate SDM performance given the validation dataset obtained with bioacoustics and different thresholds. Having four evaluation metrics would then be a methodological procedure.”**

   Answer: Thresholds are used to convert the continuous suitability maps generated by MaxEnt into presence-absence maps and were subjected to field validation. However, we also agree that our goal was to evaluate SDM performance given the validation dataset obtained using bioacoustics. So, we

added the following sentence to the text: "(…) aiming to the evaluate SDM's performance of using different thresholds given the validation dataset obtained with bioacoustics".

Methods

5. **"Perhaps a flowchart with the steps and setting used to build models could be provided to have a clearer picture of the distinct models."**

   Answer: We added a new figure (Fig. 2) with a flowchart summarizing the methodology used in this study.

6. **L. 131-132: "could you provide, in few words, how does the regularization multiplier parameter work?"**

   Answer: We added the following sentence to the text: "The regularization multiplier is a tunning parameter used to smooth the distribution prediction of the model, making it more regular and less overfitted."

7. **L. 167: "The validation points seem to be more concentrated toward east, not randomly distributed across the 1000 x 1000 km area. This pattern is similar to the historical records, which may likely be associated with accessibility."**

   Answer: Yes, in fact, road accessibility was a pre-condition for the point selection, and we failed to be clear about that. Therefore, we changed the text to: "Considering those species' potential distribution covered extensive areas, we focused our field validation on 129 randomly-selected sampling points along an area of 758,193 km^2, in the Northeastern part of Brazil (Fig 1, and S3 Table supplementary material). Since some Northeastern Brazil areas are not easily accessed, we pre-imposed the point selection near roads or paths accessible by, at least, an off-road vehicle.".

8. **L. 181: "Did the acoustic monitoring took place in all months from march 2014 – January 2020? Can you provide some information about the seasonal activity of these species?"**

   Answer: No, it did not, the acoustic monitoring took place between those dates. The seasonality in the Neotropics is defined by the differences in precipitation and the period of the year for that seasonal precipitation also differs between regions. Therefore, not only due to the size of the sampling area but due to the climate specificities of each region/biome, it would be impractical to collect acoustic data in all points simultaneously. To avoid any bias due to the sampling scheme, we chose to sample all points for at least 2 nights during its dry season, avoiding nights with rain, strong winds, or less than 15ºC, as stated in the manuscript.

   There is little information about the neotropical bats' seasonal activity, and the majority of the studies available were conducted in the Amazonia biome and/or used mist-nets. Since we did not conduct our study in the Amazonia biome, and mist-net studies are biased towards the Phyllostomidae bats;

probably, seasonal activity information available might not apply for the Caatinga, Cerrado, or Atlantic Forest populations.

Therefore, to avoid confusion and further incrementing the already long text in the methods section, we prefer not to add the seasonal activity information here.

9. **L. 182: "In line 71 you mention 300,000 files. Are these files subsets within the total continuous files? If so, please, describe how subset files were selected."**

   Answer: Yes, those files are subsets. We changed the text to: "The acoustic sampling performed in this study resulted in more than 1.5 TB of raw sound files. Those raw sound files were divided into 15-sec files and subsequently found that more than 300,000 of those 15-sec sound files contained bat calls."

"Results"

10. **"You could add a paragraph summarizing the main results found in SDM with the different settings used in the models, before validation."**

    Answer: We added a new figure with a model generated for the species *Promops nasutus* and the differences on the predicted occurrence after applying the three studied thresholds (Fig. 4).

11. **"What are the differences in performance found for different regularization multiplier values?"**

    Answer: The usage of different regularization multipliers is essential to avoid model overfitting. Therefore, we employed it as part of the modeling process and not as a feature to be validated. We did not systematically test regularization multiplier values' performance, and we believe that the best models can be found in any of them. For example, if you consult the S4 table, the best performance model for *Noctilio leporinus* (in terms of G-mean) was built with a 3.0 regularization multiplier (Reg 3), but the second-best was built with a 1.0 regularization multiplier (Reg 1). In the case of *Promops centralis*, the best performance model (in terms of G-mean) was built with a 1.0 regularization multiplier (Reg 1), but the second-best was built with a 2.0 regularization multiplier (Reg 2). There is no 'most suitable' regularization multiplier but is crucial to model using different regularization multipliers to find the best model.

12. **"L. 236: It would be nice to see an image of the distinct calls found in such an amazing acoustic dataset."**

    Answer: We added a new figure (Fig. 3) with a spectrogram displaying calls from the six studied bat species.

13. **"Figures: It would be easier to see the graphs if you provide a single panel summarizing all seven figures 2-8, where each line could be a metrics (accuracy, precision, etc.) for the distinct thresholds, and boxplots for each species would be different colors."**

Answer: Before submitting the manuscript, we thought of a solution similar to the one suggested. However, the resulting figure was too overloaded and more challenging to interpret because of the large amount of information. Therefore, we prefer to maintain all seven figures (now Figs. 5-11) instead.

“Discussion”

14. **“The first paragraph still lacks a clear summary of the main findings and a take-home message. For instance, it remains unclear what is behind the differences among the three thresholds used in this study. What are the fundamental differences between them, and which would theoretically provide a more informative result? It would also be interesting if you could discuss which models had best performance and if post-validation performance of these specific models had high and positive correlation.”**

    Answer: We changed the first paragraph to make our main findings clearer. The main differences between the three tested thresholds are explained in materials and methods, and we present the validation results of each in the “Thresholds vs. validation” results section, and we discuss extensively its performances in the discussion.

15. **“Correlation scores in table 1 are not strong, with most <0.5. This finding should be highlighted and discussed.”**

    Answer: We followed the Reviewer 2 suggestion on the problem of using Spearman Rank test correlation in our case. Therefore, to assess the performance of the theoretical evaluation metrics (OAcc, P-kappa, TSS, and SEDI), now we performed mixed-effects linear models to evaluate the correlations between those scores and the post-validation performance metrics scores obtained using acoustic monitoring (accuracy, precision, sensitivity, specificity, g-mean, and f-score), where species were treated as a random-effect variable.

16. **“Also, it would be interesting to know if the performance metrics are similar to studies that rely upon non-acoustic methods for validation.”**

    Answer: We also agree that it would be interesting. However, we think that this would need to be addressed in a new study since we would need to use independent datasets (with and without acoustic methods).

17. **“L. 374-375: This is not entirely true for all species/thresholds evaluated and could be acknowledged here.” In this first paragraph, you could be more specific in the take-home message. For instance, you could include information on how different thresholds may be better than others and discuss the influence of sample size and unbalanced data.**

    Answer: We stated: “Bioacoustics proved to be a very effective method for the *in situ* validation of SDM for six neotropical bat species in a large and poorly-sampled area in Brazil.” We still think that

bioacoustics proved to be a very effective validation method of SDM's since it allowed us to easily flag SDM's omission and commission errors in a vast study area with a relatively low sampling effort. After your and Reviewer 2's remarks, we linked our statements with our results and we edited and reviewed entirely this first paragraph. We discuss the influence of the threshold choice and sample size on the "Theoretical model evaluation metrics and thresholds vs. validation" section of the discussion.

Reviewer 2' comments

This study examines the contribution of bioacoustic tools (passive acoustic monitoring) to validate the predictions of Species Distribution Models (SDM) in six tropical bat species. Based on the comparison between theoretical evaluation metrics and post-validation performance parameters obtained from field sampling, the authors highlight the need of in situ validation of SDM and argue the use of novel acoustic techniques as rapid validation methods. The study has been properly conducted, using sound methods and a large data set, which enable the authors to successfully address the proposed goals. Overall, the manuscript is clear, well-written and presents results in an effective manner. Nevertheless, there are still a series of issues that should be carefully revised before publication. First, I strongly recommend an English revision of the whole text by a native speaker or language service, if it has not been made yet. I am not an English native speaker, but I feel this is needed to significantly improve spelling, grammar, and the general flow of the text. All across the manuscript, I included suggestions and minor questions (directly on the pdf; see attachment) that aim to increase the clarity and precision of the document. My major points are listed below.

"Statistical analysis"

1. **"My main concern is related to the statistical analyses, since some of them may be fell into pseudoreplication. As shown in Table 1, Spearman correlation tests were calculated using 144 observations (except for SEDI) that came from models of the six study species (24 models per species). Thus, these observations (validation metrics) are grouped by species and they must be not considered as independent replicates. Predictions obtained by models of the same species can likely be related. As consequence, the statistical analysis applied to examine the correlation between theoretical evaluation and in situ validation should be designed taking into account the non-independence of the observations and performed again. I recommend the use of general linear mixed-effects models (GLMM), for instance, with species as random factor. Probably, the same might be applied to Kruskal-Wallis tests that the authors should carefully review in the light of this comment about potential pseureplication."**

   Answer: We believe that our design is free from pseudoreplication since sampling points are apart at least 2 km from each other. Therefore, is not possible to record the same bat at the same time in different sampling points. We replaced the Spearman's Rank correlations with we performed mixed-

effects linear models to evaluate the correlations between those scores and the post-validation performance metrics scores obtained using acoustic monitoring (accuracy, precision, sensitivity, specificity, g-mean, and f-score), where species were treated as a random-effect variable. However, we maintained the Kruskal-Wallis tests since we believe our data is pseudoreplication-free.

“Results interpretation, conclusions and goals”

2. **“Despite of the fact that the manuscript is generally well-written and structured, I find that the link between results and discussion is still unclear, especially for a general audience with less experience on SDM. In results, the reader can find vast details and analyses, but there are not clear explanations about the implications of these findings. In discussion, the interpretation of the results is often presented in a general manner, hindering the general understanding of the origin of such conclusions. I recommend the authors to make an effort to clarify (in results or discussion; or even better, in both) which specific result in each case enable them to draw a particular conclusion, so that the text gain in clearness and can be accessible for a broader audience. Which specific result helps us to understand that validation is key for SDM? Which one indicates that bioacoustics is “a very effective method for the in situ validation of SDM”? Which one that “we empirically demonstrated that independent field surveys are the best approach to corroborate the predictions made by modelling”?”**

   Answer: We supported the highlighted conclusions with some examples extracted from our results, as suggested by the reviewer.

3. **“Moreover, in my opinion, the text would also benefit from a more clear link between the goals presented in the last paragraph of the introduction and the main conclusions presented in the first paragraph of the discussion. The paragraph presenting the study goals lacks an explicit mention to the general aim of the study. The authors did not clearly refer to a key aspect of the study: the assessment of the role of validation methods in SDM and their proposal of using bioacoustic tools as rapid validation method.”**

   Answer: We agree and we corrected it, adding two sentences in the paragraph presenting the study: “(…) aiming to the evaluate SDM’s performance of using different thresholds given the validation dataset obtained with bioacoustics” and “This methodological procedure enabled us to assess of the role of validation methods in SDM outputs and acoustic samplings as rapid validation method.”. We also reviewed and edited the text to add a clear link in the discussion to each of the goals presented in the conclusions.

“Methodological aspects that also require clarification”

4. **“Historical species records**

Authors conducted a literature review for gathering distribution records of the study species. However, some details are missing and prevent the reader from properly understand how this review was carried out. Did authors use keywords in databases as Google Scholar, WoS or Scopus? Did they refine the search by specific field areas? How many documents met the criteria and were reviewed? How many were used to determine the historical records?"

Answer: Yes, we used keywords. We chose to omit this part due to the already large methodology section of this manuscript. However, we agree with the reviewer that, in this way, the review we carried out would be difficult to understand and would hinder the reproducibility of this study. Therefore, we included the following sentences:

- "For the conducted review, we searched for publications using keywords such as: "neotropical bats"; "Pteronotus"; "Noctilio"; "Promops"; "Saccopteryx"; "Pteronotus personatus"; "P. personatus" and so on. We did not refine the search by specific field areas nor geographical areas."
- "We only selected bat records from peer-reviewed literature, books, or online databases supported by voucher specimens."

5. **"Distribution modelling procedure**

   This section is well-written, clear and full of details. I particularly appreciate the modelling design applied by the authors that took into account a large number of factors and criteria, and performing a diversity of models. I only have a few minor questions (see the pdf) and a major one: While historical records are taken as presences, how did the authors treat absences and pseudo-absences in the SDM models? I think this should be clarify, considering the significant knowledge shortfalls in species distribution, and the relevant effect of absence in SDM models."

Answer: We agree that absences and pseudo-absences are a significant shortfall and can have a relevant effect on SDM. This was one of the main reasons for our modeling algorithm software choice since MaxEnt uses presence-only input data. To clarify our preference for MaxEnt, we added the following sentence to the manuscript: "Due to the shortfalls and constraints of absences and pseudo-absences in the species distribution knowledge, and consequently, in species distribution models, we chosen MaxEnt as it uses presence-only input data, can include both categorical and continuous covariables, and create a spatially explicit suitability map for the focal species."

6. **"Acoustic sampling**

   A large number of details and information should also be added in this section to properly describe some key points (see comments on pdf). Particularly, a key assumption of the study is that the number of sampled days was large enough to get representative data of bat activity in each site. How can we be sure that sampling a

minimum of two days enable the authors to determine species presence in a given location?"

Answer: Despite *Promops centralis* was recently listed in the study area in a recent study (see Hintze et al. 2020, DOI: 10.1093/jmammal/gyz167) using acoustic samplings, all six chosen bat species are considered widespread in the study area and easily recorded by acoustics. We could have added more species to the study but some are restricted to an area/biome (e.g., *Molossops temminckii*, *Pteronotus rubiginosus*, *Peropteryx kappleri*, etc.) and/or their calls are more difficult to record due to sound dissipation (e.g., *Rhychonycteris naso*, *Natalus macrourus*, etc.). In the points where we identified each of the six selected species, we detected at least one echolocation sequence right on the first night. However, we preferred to be conservative, and despite we have more than 129 points with acoustic information, we only used those with at least two complete nights sampled. Like all other sampling methods, species completeness is very hard to achieve in rapid samplings. But to cover a wide area such as ours, and with a limited number of recording devices as we had, it is logistically impossible to record for longer periods. The number of sampled nights varied from 2 to 15 nights.

Reviewer 2's comments on the manuscript

7. **L. 15: "it is a..."**

   Answer: Corrected as suggested.

8. **L. 16-17: "too obvious. as any other method"**

   Answer: Sentence removed.

9. **L. 22: "I think this is an overstatement. Although the area drawn between the farthest is that big, this is not the area "covered" by your monitoring program, as there are considerable gaps within the area. I would say that the recorders were deployed over that area... but they are not "covering" that area, as their "detection space" are not registering bat activity over the 758.000 km^2."**

   Answer: Sentence changed to "Here, we used extensive acoustic monitoring (>120 validation points over an area of >758,000 km^2, and producing >300,000 sound files) to validate MaxEnt outputs for six neotropical bat species in a poorly-sampled region of Brazil."

10. **L. 42: "I would say "... gained importance worldwide in the .... and conservation.""**

    Answer: Sentence changed as suggested.

11. **L. 45: "estimates of what?"**

    Answer: Sentence changed to "This is an advantage for large-scale approaches and species distribution estimates since few parts of our planet have been adequately sampled".

12. **L. 45: "the planet"**

    Answer: Sentence changed to "This is an advantage for large-scale approaches and species distribution estimates since few parts of our planet have been adequately sampled".

13. **L. 45: “have been”**

    Answer: Corrected as suggested.

14. **L. 49: “This is true for any application of SDM. As any other method, it is expected that SDM be reliable for whatever purpose”**

    Answer: Sentence changed to “For biodiversity conservation purposes, imprecise models can undermine the calculation/estimate of a species' occupancy, a criterion used to assess its conservation status, for example”.

15. **L. 51: “Consider rephrase. The sentence begins by "for instance" and ends by "for example"**

    Answer: Sentence changed to “For biodiversity conservation purposes, imprecise models can undermine the calculation/estimate of a species' occupancy, a criterion used to assess its conservation status, for example”.

16. **L. 52: “the model predictions may not forecast...”**

    Answer: Corrected as suggested.

17. **L. 54: “the potential distribution of a given taxon"**

    Answer: Corrected as suggested.

18. **L. 57: “why is this urgent in some cases? Be explicit and explain this point”**

    Answer: We changed the sentence to “Therefore, in situ validation of the SDM outputs should be a critical step – in some cases, urgent [10, 11] since unvalidated species potential distribution maps can influence and hinder species assessments and the decision-making for species conservation.”

19. **L. 61: “The order seems to me a bit odd”**

    Answer: Sentence changed to “Bioacoustics is one of such techniques and has been used for a long time to record species presence/absence for amphibians, birds, and cetaceans”.

20. **L. 64-65: “can be easily recorded thanks to...”**

    Answer: Corrected as suggested.

21. **L. 70-71: “I think this is an overstatement. Although the area drawn between the farthest is that big, this is not the area "covered" by your monitoring program, as there are considerable gaps within the area. I would say that the recorders were deployed over that area... but they are not "covering" that area, as their "detection space" are not registering bat activity over the 758.000km^2.”**

    Answer: Sentence changed to “Here, we used an extensive in situ monitoring (>120 validation points over an area of >758,000 km^2, and producing >300,000 sound files) of echolocation calls (…)”

22. **L. 80: “It can be omitted”**

    Answer: Omitted as suggested.

23. **L91: “it should be out of the link”**

    Answer: Corrected.

24. **L. 108: “WorldClim Which version? resolution? Which specific variables? All bioclimatic ones?”**

Answer: Sentence changed to “We used SDMtoolbox 2.4 for ArcGIS [23] to create an environmental heterogeneity map with all bioclimatic variables from WorldClim 2.0 [24].”

25. **L. 121: “I did not understand this point. Which is the categorical variable? Consider to rephrase.”**

    Answer: Sentence changed to “We used the 19 bioclimatic variables plus elevation available at the Worldclim data website [24], and Globcover 2009 [30] as a categorical variable for land cover.”

26. **L. 136: “bioacoustical or bioacoustic. "bioacoustics" is the science, while "bioacoustic" is the adjective”**

    Answer: Changed to “bioacustical” as suggested.

27. **L. 158: “validated in the field”**

    Answer: Changed as suggested.

28. **L. 159: “for”**

    Answer: Changed as suggested.

29. **L. 162: “As the next section is "model validation", I suggest this to be termed as "acoustic monitoring and species identification", or something similar”**

    Answer: Subtitle changed to “Acoustic monitoring and species identification” as suggested.

30. **L. 163-164: “Where did these species distributions come from? Please specify how they were obtained”**

    Answer: When we use MaxEnt to model species distribution, we also obtain a model output that is the average of all models performed. To be more explicit, we changed the sentence to: “For the selection of the sampling points for field validation, we summed the MaxEnt’s given average potential distribution outputs of the six species to identify regions with the highest and lowest suitability of species occurrence, but without historical records.”

31. **L. 168: “Again”**

    Answer: Corrected as described in point 21 of this list.

32. **L. 171: “It would be useful to add a general description of the sample sites (habitat types, overall characteristics, etc.)”**

    Answer: We added the biome, TNC ecoregion and globcover land cover description for all sample sites in a new version of S3 Table.

33. **L. 172: “How and where these recorders were deployed and installed? Please describe the procedure. How many recorders were used in total of each type?”**

    Answer: To insert the requested information in the text, we changed the sentence to “Between March 2014 and January 2020, we employed passive acoustic monitoring to sample bat echolocation calls in the 129 sampling points (Fig 1, and S3 Table in supplementary material), using a combination of two SM2Bat+, two SM3BAT, and two SM4BAT-FS ultrasound recorders (Wildlife Acoustics Inc., Massachusetts, USA). We set the microphones at 45º to the ground, avoiding highly cluttered areas [16, 44, 45].”

34. **L. 176: “Please indicate also audio bit depth, audio format and number of channels”**

Answer: To insert the requested information in the text, we changed the text to "Since the highest frequency used by the studied species is ~60 kHz (*Noctilio leporinus*) [18], we configured the bat detectors with a minimum sampling rate of 384 kHz and 16 bit audio depth, enough to detect and record our focal species without distortions (e.g., aliasing). Each sampling point was acoustically monitored for at least two nights (from 2 to 15 nights), recording continuously from 30 minutes before sunset until 30 minutes after sunrise. The recordings were stored automatically in mono .wav format in the SD cards with a preset maximum duration of 1 minute if any sound above 7 kHz exceeded at least 6 dB."

35. **L. 176: "Could you also add a range and/or an average of the number of sampled days per site? So that we get an idea of maximum or mean number of days"**

    Answer: We added the requested information to the text, please see the point above.

36. **L. 177: "On the section Results, authors describe a number of files. Please define how long each file was or the recording schedule used to program acoustic sensors"**

    Answer: We changed the text to include this information, please see above in point 34 and below in point 39.

37. **L. 180-181: "I suggest to place this information above in the paragraph together with additional details about the installation of the recorders in the field"**

    Answer: We moved the sentence as suggested.

38. **L. 183: "It can be omitted, it is pretty obvious"**

    Answer: Omitted as suggested.

39. **L. 184: "First it should be described how authors found those sequences across the time-series of recordings. Did you visually inspect spectrograms? Did you use a automated recognition method? Did you sub-sample the hours of recordings obtained in each site?"**

    Answer: To insert the requested information in the text, we changed the text to "We used Raven Pro 1.5 (The Cornell Lab of Ornithology 2014) for the acoustic analysis. To ease the acoustic analysis, we were divided the raw 1-minute sound files in 15-sec files and, subsequently, we visually inspected all files for the desired bat species calls after configuring the spectrograms to DFT equals 1024, 96% overlap, window length to 1 ms, using Hamming windows. We only analyzed sequences containing a minimum of three search calls with a good signal-to-noise ratio (> 15 dB) [46, 47]. We performed manual acoustic identification using qualitative (e.g., call structure and modulation) and quantitative parameters (e.g., frequency of maximum energy, maximum and minimum frequency, call duration, etc.), following previously published studies on neotropical bat acoustic identification [e.g., 17-19, 47, 48, 49]."

40. **L. 192: "their calls"**

    Answer: Changed as suggested.

41. **L. 197: "Model validation or Field validation of the models"**

    Answer: Subtitle changed to "Field validation of the models" as suggested.

42. **L. 203-204: “To the best of my knowlege, the type errors are swapped: FN = Type II error FP = Type I error”**

    Answer: Yes, the type errors are swapped. We corrected in the text.

43. **L. 210: “remove”**

    Answer: Removed.

44. **L. 212: “remove”**

    Answer: Removed.

45. **L. 212: “"with high g-mean indicating..."”**

    Answer: Changed as suggested.

46. **L. 213: “* or x”**

    Answer: Changed to *.

47. **L. 216: “* or x”**

    Answer: Changed to *.

48. **L. 223: “I suggest to indicate the origin of these metrics. For instance "... scores obtained using acoustic monitoring" or something similar”**

    Answer: As requested, we changed the text to “(...) and the post-validation performance metrics scores obtained using acoustic monitoring (accuracy, precision, sensitivity, specificity, g-mean, and f-score).”

49. **L. 224: “Describe and justify here the use of Bonferroni correction included in results”**

    Answer: We used Bonferroni correction to reduce the probability of having false positive statistical significant correlations. However, as suggested, in this new version of the manuscript we employed mixed-effects linear models to evaluate the correlations between those scores and the post-validation performance metrics scores obtained using acoustic monitoring (accuracy, precision, sensitivity, specificity, g-mean, and f-score), where species were treated as a random-effect factor.

50. **L. 230: “SDMs or remove it, so that it reads "acoustic SDM validation"”**

    Answer: We changed to SDMs as requested.

51. **L. 231: “acoustical or acoustic "acoustics" is the science, while "acoustic" is the adjective”**

    Answer: We changed to “acoustic”.

52. **L. 232: “how long are these sound files? 1-min files, 5-min, 1h,...?”**

    Answer: To insert the requested information in the text, we changed the text to “The acoustic sampling performed in this study resulted in more than 1.5 TB of raw 1-minute sound files. Those raw sound files were divided in 15-sec files and subsequently found that more than 300,000 of those15-sec sound files contained bat calls.”

53. **L. 254: “I think a single time is enough to cite the Table 1 in this paragraph”**

    Answer: Done.

54. **L. 260: “bioacoustical or bioacoustic”**

    Answer: Changed to “bioacoustic”.

55. **L. 267: “here and in all the paragraph: "averaged" or "mean"”**

Answer: We changed to "averaged" and applied to all the paragraph.

56. **L. 268: "here and in all the paragraph: Consider to describe using a more precise expression, such as "P10-based models" or "P10-based predictions"..."**

    Answer: We changed to "P10-based models" and applied to all the paragraph.

57. **L. 270: "in"**

    Answer: Changed.

58. **L. 270: "omit"**

    Answer: Omitted.

59. **L. 271: "Consider to rephrase"**

    Answer: We rephrased to "P10-based models obtained overall averaged sensitivity scores higher than maxSSS but lower than LPT-based models and specificity scores higher than LPT but lower than maxSSS-based models (Fig 5, and S6 Table in supplementary material)."

60. **L. 285: "remove"**

    Answer: Removed.

61. **L. 378: ""rich in bat species""**

    Answer: Changed as requested.

62. **L. 381: "remove"**

    Answer: Removed.

63. **L. 383-384: "What are the data or results that allow to state this conclusion? please refresh here (maybe also in results) so that you help the reader to understand where this statement comes from"**

    Answer: We added this text to the manuscript: "We demonstrated that only using pre-existing data subsets, our model evaluation may be restricted to the theoretical evaluation metrics particularities and existing data constraints, and selecting the highest score model may not mean that we chose the model that represents the reality better. A *Noctilio leporinus*' maxSSS-based model was a great example since notwithstanding obtaining the best TSS score for the species models, this model also displayed very high omission errors (>30%) when we validated it in the field. Thus, high theoretical evaluation metric' scores such as the widely used TSS may guarantee reliable models but could not provide the best model for our purposes and may also tend to score higher for the most overfitted models. This problem can be exacerbated when dealing with species with fewer existing data records, such as *Promops centralis*."
