# Supplementary material for: Bioacoustics for in situ validation of species distribution modelling: An example with bats in Brazil
Source: PLoS One. 2021 Oct 20;16(10):e0248797. doi: 10.1371/journal.pone.0248797 (PMC8528307; doi:10.1371/journal.pone.0248797)
Supplement: Attachment — Submitted filename: Response to Reviewers_Rev2.pdf [file pone.0248797.s009.pdf]

Centro de Biociências **Universidade Federal de Pernambuco**

From: Frederico Hintze
To: Dr. Daniel de Paiva Silva – Academic Editor, PLOS One
Ref.: PONE-D-21-07379

Recife, October 5th 2021.

Dear Dr. Daniel de Paiva Silva,

I would like to resubmit for possible publication in PLOS One our manuscript "Bioacoustics for in situ validation of species distribution modelling: An example with bats in Brazil", PONE-D-21-07379, whose authors are Ricardo B. Machado, Enrico Bernard, and myself as first author.

Thank you very much for the review of the manuscript, the comments and suggestions you made. We carefully reviewed and addressed all notes and change proposals made by you and the reviewers. Below you will find a detailed list of comments and corrections made based on the points you and reviewers raised. Please let us know if any other information is necessary.

Best regards,

Frederico Hintze
Laboratório de Ciência Aplicada à Conservação da Biodiversidade
Departamento de Zoologia, Universidade Federal de Pernambuco
Rua Professor Nelson Chaves s/n, Cidade Universitária, Recife, PE 50670-420 Brazil
Phone/fax (+55) 81 2126 8353 e-mail: fredhintze@gmail.com

Academic Editor's comments

Please review your reference list to ensure that it is complete and correct. If you have cited papers that have been retracted, please include the rationale for doing so in the manuscript text, or remove these references and replace them with relevant current references. Any changes to the reference list should be mentioned in the rebuttal letter that accompanies your revised manuscript. If you need to cite a retracted article, indicate the article's retracted status in the References list and also include a citation and full reference for the retraction notice.

Answer: We reviewed our reference list and text citations, and we did not find any missing references or citations neither any retracted papers.

Additional Editor Comments

Dear Hintze et al,

After a new review round, I must say that you are almost there. One of the original reviewers decided for the acceptance of your manuscript, whereas the other decided for a minor review. Therefore, I will grant you all a one-month period in order to proceed with the necessary changes. In case you need more time, please let me know. Still, do not hesitate to submit earlier then the estipulated deadline in case you can.

Best regards,

Daniel Silva, PhD.

Answer: We reviewed and addressed all change proposals made by the reviewers. Below you will find a detailed list of comments and corrections made based on the points reviewers raised.

Reviewer 1’ comments

I thank the authors for addressing the previous suggestions. This is an interesting contribution combining SDM and bioacoustics, and I congratulate the authors for their work. Finally, I would just suggest adjusting some minor concerns, detailed below.

Answer: We thank you for all pertinent comments and suggestions throughout this review, which added clarity and quality to our manuscript.

Reviewer 1’s comments on the manuscript

1. **Figures 5-11: I think that you should reconsider joining all boxplots in a single figure. I suggest that you use accuracy, precision, sensitivity, specificity, g-mean and f-score as lines, and for columns, use LPT, maxSSS, and p10 for repeated for each species. Such as:**

   Species 1 / Species 2 / Species 3 …
   LPT. maxSSS P10 / LPT. maxSSS P10/ LPT. maxSSS P10
   Acc
   Pre
   Sen
   Spe
   Gme
   Fsc
   Species 6 / Species 7 / Species 8…
   LPT. maxSSS P10 / LPT. maxSSS P10/ LPT. maxSSS P10
   Acc
   Pre
   Sen
   Spe
   Gme
   Fsc

   Let the first row with 4 species and the second row with 3.

   Answer: We reconsidered and, as suggested, we replaced former figures 5-11 for a single figure (Figure 5).

2. **424: “we proved” is not exactly what you show here, since the performance of the validation is subject to interpretation and depends on many factors, as tuning parameters and thresholds. I believe that the message should be to explore variations in modeling approaches using SDM, since predictions are largely variable even for approaches that are considered optimum.**

   Answer: With this sentence, we meant that we confirmed bioacoustics are valuable as a field validation method of SDM’s in the case of bats in a large and sub-sampled area. We also agree with you on the missing message. Therefore, we added the following sentences to the manuscript (Lines 400-404): “Species distribution modeling is subject to interpretation and depends on many factors, such as tuning parameters and thresholds [7, 8, 29, 36, 38]. After using field validation, we urge

modelers to explore their modelling approaches effects on result models since predictions might be very variable even when applying optimal practices.”

Reviewer 3’ comments

I greatly enjoyed reading this paper, and believe it constitutes an important contribution to the literature on species distribution modeling (SDM), especially in Neotropical region, particularly as it provides important insights on the validation process of SDM. The article is interesting and overall reads well; however, I have minor comments. The validation approach (based on acoustics) is especially important for non-phyllostomid bats since bats from the Phyllostomidae family have considerable overlap in their call parameters (Yoh et al. 2020). In that sense, I suggest clarifying the main target bat species, which acoustics as validation method, is proper.

Answer: We are thankful for your comments and suggestions. We also agree that the overlapping of the acoustic parameters of the vast majority of Phyllostomidae does not allow acoustical methodologies for studying this same family. For this reason, we are changing our text clarifying this matter.

1. **Is there any season effect on acoustic surveys explaining absences and potentially affect validation?**

   Answer: Our acoustic monitoring took place between March 2014 and January 2020. The differences in precipitation define the Neotropics' seasonality, plus the period for that seasonal precipitation also differs between regions and biome. Therefore, due to the sampling area size and the climate specificities of each biome, it would be impractical to collect acoustic data in all points simultaneously. However, to avoid any bias due to seasonality, we sampled all points for at least two nights during its dry season. As stated in the manuscript, we avoided sampling at nights with rain, strong winds, or less than 15ºC.

   Moreover, there is little information about the neotropical bats' seasonal activity: most of the studies available on the subject were conducted in the Amazonia biome and/or used mist-nets. Since we did not perform our study in the Amazonia biome and mist-net studies are biased towards the Phyllostomidae bats, seasonal activity information might not apply to the Caatinga, Cerrado or Atlantic Forest populations. Therefore, to avoid confusion and further incrementing the already long text in the methods section, we prefer not to add the seasonal activity information here.

Reviewer 3’s comments on the manuscript

2. **Lines 80 – 82: It is true for several families, but it is problematic for Phyllostomidae (Yoh et al. 2020). I suggest clarifying this limitation.**

   Answer: We changed the sentence to “Moreover, with the exception of the Phyllostomidae family, most bats have conspicuous and species-specific calls, allowing precise identification of the emitter's identity and making the record of its presence accurate”.

3. **Lines 428 – 431: It is not true for all bats**

Answer: In our sentence, we stated: “For species with conspicuous vocalizations (...)”. So, we believed that it was implicit that some species do not have conspicuous vocalizations. However, to make it explicit, we changed the portion of the sentence “(...) like bats (...)” to “(...) like most bat families (...)”.

Therefore, now we state: “For species with conspicuous vocalizations – like most bat families – the acoustical samplings we employed showed the potential to better refine SDMs in large and under-sampled regions, with relatively low sampling effort.”.
